# Painting Materials and Technique for the Expression of Chinese Inheritance in Liu Kang’s Huangshan and Guilin Landscapes (1977–1996)

**DOI:** 10.3390/ma15217481

**Published:** 2022-10-25

**Authors:** Damian Lizun, Teresa Kurkiewicz, Bogusław Szczupak, Jarosław Rogóż

**Affiliations:** 1 Heritage Conservation Centre, National Heritage Board, 32 Jurong Port Rd, Singapore 619104, Singapore; 2 Department of Painting Technology and Techniques, Institute for Conservation, Restoration and Study of Cultural Heritage, Nicolaus Copernicus University, ul. Sienkiewicza 30/32, 87-100 Toruń, Poland; 3 Department of Telecommunications and Teleinformatics, Wrocław University of Science and Technology, Wybrzeże Stanisława Wyspiańskiego 27, 50-370 Wrocław, Poland

**Keywords:** Liu Kang, pigment identification, SEM-EDS, FTIR, IRFC, PLM, X-ray, hidden paintings

## Abstract

Liu Kang (1911–2004) was a Chinese artist who settled in Singapore in 1945 and eventually became a leading modern artist in Singapore. He received academic training in Shanghai (1926–1928) and Paris (1929–1932). Liu Kang’s frequent visits to China from the 1970s to the 1990s contributed to a special artistic subject—the Huangshan and Guilin mountains. This subject matter triggered an uncommon painting approach for his oeuvre. In this context, this study elucidates the artist’s choice of materials and methods for the execution of 11 paintings, dating between 1977 and 1996, depicting Huangshan and Guilin landscapes. The paintings belong to the collection of the National Gallery Singapore. They were investigated with a combination of non- and micro-invasive techniques, supplemented by a wealth of documentary sources and art history research. The obtained results highlight the predominant use of hardboards resembling Masonite^®^ Presdwood^®^ without the application of an intermediate ground layer. Commercially prepared cotton and linen painting supports were used less frequently, and their structure and ground composition were variable. This study revealed the use of a conventional colour base for the execution of the paintings—a consistent colour scheme favouring ultramarine, yellow and red iron-containing earths, viridian and titanium white. Less commonly used pigments include Prussian blue, cobalt blue, phthalocyanine blue, phthalocyanine green, naphthol red AS-D, umber, Cr-containing yellow(s), cadmium yellow or its variant(s), Hansa yellow G, lithopone and/or barium white and zinc white and bone black. The documentary sources indirectly pointed to the use of Royal Talens, Rowney and Winsor & Newton, brands of oil paints. Moreover, technical and archival findings indicated the artist’s tendency to recycle rejected compositions, thereby strongly suggesting that the paintings were executed in the studio. Although this study focuses on the Singapore artist and his series of paintings relating to China, it contributes to existing international studies of modern artists’ materials.

## 1. Introduction

Born in China, Liu Kang (1911–2004) is considered one of Singapore’s leading modern artists. His stylistic preferences were initially shaped by the art training obtained from Xinhua Arts Academy in Shanghai, followed by studies at the Académie de la Grande Chaumière in Montparnasse, which exposed him to the Impressionism and Post-Impressionism art movements [1]. However, Liu Kang’s personal and mature approach to painting was strongly influenced by his regional travels [2]. The most remarkable was a trip to the Indonesian island of Bali in 1952, which inspired him to contribute to the formulation of a new painting concept known as the Nanyang style. The style depicted aspects of the Southeast Asian way of life, integrated Western and Chinese means of artistic expression and incorporated stylistic elements of the batik technique [3,4,5,6]. Due to growing nostalgia for the motherland, China was Liu Kang’s most often visited country since the communist state opened up to the world in the 1970s [2,7]. Trips to China resulted in countless drawing and painting studies of the majestic landscapes of Huangshan and Guilin mountains [1]—places he remembered from his stay in Shanghai [8]. In a 1955 interview, he recalled: “Every artist should some day visit those mountains. They have been painted by the most famous Chinese masters and have exerted a great influence on the style of Chinese painting. We can learn a great deal from Nature—forms, colours, compositions and moods—and the Yellow Mountains [Huangshan] are inspiring” [9]. Liu Kang’s memories of Chinese nature were still vivid in 1969 when he wrote in his essay: “Mount Huang can be considered an exemplification of all the best elements of Chinese landscape. However, the most important thing is that once a person falls into its embrace, he naturally feels that everything is spacious and grand, lofty and vigorous, and his mind is infinitely expanded” [10].

The subsequent visits to China from the 1970s onwards revealed Liu Kang’s continued fascination with the country’s nature, which remained the source of his artistic vision for many years [11]. Moreover, these visits reinforced the artist’s sense of Chinese identity, which he expressed in 1993: “Legally, I am a Singapore citizen, but I am Chinese by birth. The Chinese have a long history of several thousand years with deep and firm cultural roots and a distinctive artistic achievement. Besides, she has a vast territory with a magnificent landscape. That is why I have tried to reveal the robust spirit, profound contents and refined taste of her culture in my paintings” [12]. Hence, the adopted painting style and technique for depicting the mountainous Chinese landscapes radically differ from those seen in his early artistic phases associated with practices in Paris [13] and Shanghai [14], post-war Singapore [15], Nanyang style artworks [6] as well as his special theme of Nudes [16]. The ethereal style [1] of the investigated paintings conveys a unique atmosphere of the mountainous landscapes, characterised by the rough structure of the majestic rocks and heavy clouds, which contrasted with delicate vegetation and moisture that obliterates the shapes of the mountains. These effects were achieved with a rich texture and exceptionally heavy impastos, which were uncommon in Liu Kang’s general painting technique. Moreover, the artworks strike the viewer with a restricted palette of colours—an unusual choice for the artist known as a skilful colourist [17,18]. These new visual effects undoubtedly reveal the strong impact that the harsh but captivating natural scene made on Liu Kang. The adopted artistic expression seems to reflect an Eastern approach to painting as described by Liu Kang in his 1969 essay: “Eastern artists treat natural scenery as the starting point for depicting their emotions” [19]. The intentional abandonment of the human element in the entire series of paintings additionally enhances the sense of isolation. Another distinguishing feature of the artist’s technique is a frequent use of hardboards, which seem to play an important role in facilitating his robust painting technique and the inconvenience of possible outdoor painting sessions. Hence, these peculiarities explain the authors’ interest in conducting a comprehensive analyses of Liu Kang’s landscapes of Huangshan and Guilin.

This investigation aims to characterize, for the first time, Liu Kang’s working process and materials employed for the painterly documentation of the mountainous Chinese landscapes, which he intensively studied from 1977 to 1996. Therefore, the development of artistic ideas, the role of the primary painting supports in the creative process, the pigments selection and the handling of the paint are thoroughly investigated. Moreover, this study expands the knowledge of Liu Kang’s style and painting practice beyond the common public perception of his close affiliation with the entire development of the Nanyang style [20,21]. Although the artist is mainly recognised in Singapore and Southeast Asia, and the studied genre relates to China, this research reaches beyond these regions by contributing to the growing knowledge of twentieth-century painting materials and their availability in Singapore.

## 2. Materials and Methods

### 2.1. Materials

**Figure 1:**
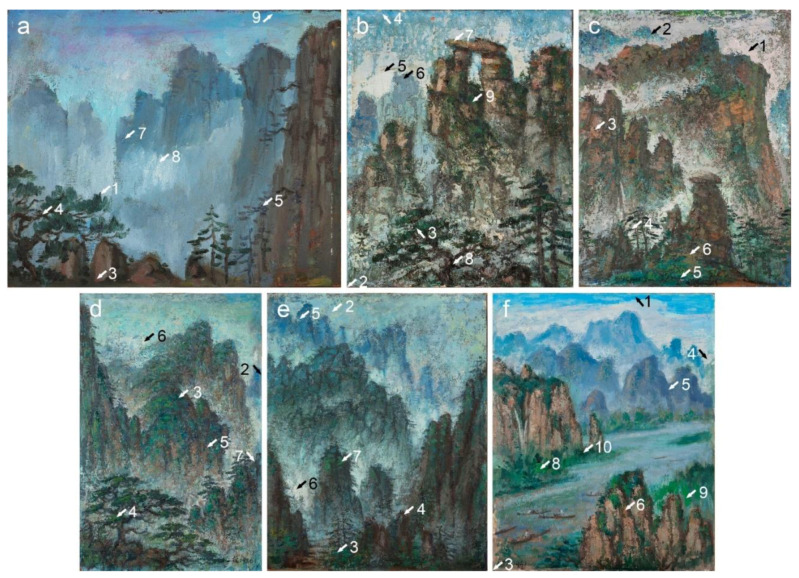
Paintings by Liu Kang: (**a**) *Mountain*, 1977, oil on board, 38 × 45.5 cm; (**b**) *Mountain*, 1981, oil on canvas, 36 × 29.5 cm; (**c**) *Mount Huangshan*, 1983, oil on board, 84.6 × 64.5 cm; (**d**) *Mount Huangshan*, 1986, oil on board, 74 × 48.5 cm; (**e**) *Mount Huangshan*, 1987, oil on board, 71 × 55.6 cm; (**f**) *Mountains*, 1991, oil on canvas, 76.5 × 61 cm. Gifts of the artist’s family. Collection of National Gallery Singapore. Arrows and numbers indicate sampling areas.

**Figure 2:**
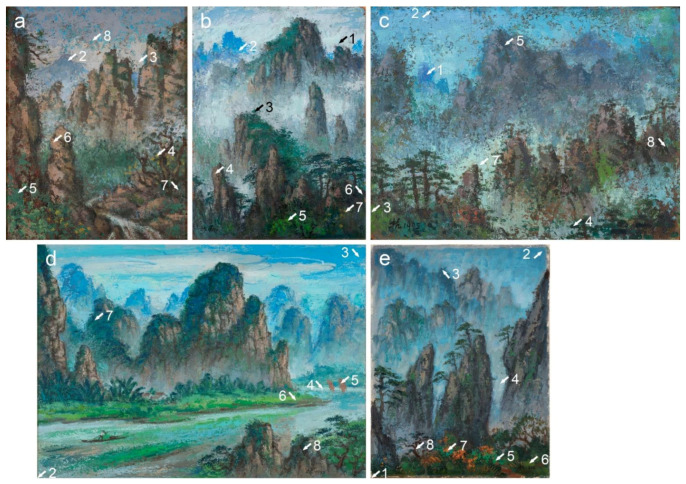
Paintings by Liu Kang: (**a**) *Mount Huangshan*, 1993, oil on board, 40.5 × 31.7 cm; (**b**) *Mount Huangshan*, 1994, oil on board, 78.5 × 58.3 cm; (**c**) *Mount Huangshan*, 1995, oil on board, 30.5 × 40.5 cm; (**d**) *Mountain*, 1995, oil on canvas, 84.7 × 118.5 cm. (**e**) *Mount Huangshan*, 1996, oil on canvas, 64 × 49 cm. Gifts of the artist’s family. Collection of National Gallery Singapore. Arrows and numbers indicate sampling areas.

The discussion of Liu Kang’s painting materials and the execution of the Mount Huangshan and Guilin landscapes is based on a technical study of 11 artworks (Figure 1 and Figure 2), spanning the period 1977–1996, from the National Gallery Singapore. It should be noted that the title of the four paintings, *Mountain(s)*, is generic and does not refer to any specific location. However, a Liu family member familiar with the artist’s trips suggested that the characteristic views of *Mountains* (1991) and *Mountain* (1995) relate to the Guilin mountains. Therefore, the remaining two paintings bearing the same title likely depict Mount Huangshan.

The research materials also included 77 paint and ground microsamples extracted from the paintings (Figure 1 and Figure 2). As seven paintings were made on hardboard and four on the canvas, only eight textile fibre samples were collected. The fibres were extracted from the threads of weft and warp of each textile. The inventory data of the paintings are summarised in Table 1.

### 2.2. Methods

The adopted research strategy relied on the methods used by the authors in the previous investigation campaigns of Liu Kang’s paintings [6,13,16]. Such an approach ensured the consistency of the acquired data and their interpretation. Firstly, non-invasive investigation techniques were carried out to collect technical data, characterise the structure of the primary supports, tentatively identify the pigments, determine the sampling areas and collect the visual evidence of the underlying compositions. These techniques involved the photography of the paintings in visible light (VIS), ultraviolet fluorescence (UVF), reflected ultraviolet (UVR), near-infrared (NIR), X-ray radiography (XRR) and surface digital microscopy. Secondly, a small number of paint fragments from each painting was extracted and prepared for the examination by several analytical techniques with the aim of determining the constituent materials of the ground and surface paint layers. These techniques comprised optical microscopy (OM), polarised light microscopy (PLM), field emission scanning electron microscope with energy dispersive spectroscopy (FE-SEM-EDS) and attenuated total reflectance-Fourier transform infrared spectroscopy (ATR–FTIR). The identified pigments were described by their common name and colour index generic name. The identification of fibres was undertaken by studies of their morphology and chemical staining tests using OM.

The interpretation of the obtained analytical data was supported by the archival sources. The artist’s studio photographs, combined with his drawings and photographs of Huangshan and Guilin landscapes from the 1970s and 1980s contribute to tracing his working process from the conception of the ideas to finalised artworks. Moreover, a few still images from the 1982 TV documentary and colourmen catalogues expanded our knowledge of the artist’s choice of painting materials and technique.

#### 2.2.1. Technical Photography

Imaging was carried out using a modified to full spectrum (360–1100 nm) Nikon 850 DSLR camera with a Nikon AF Micro NIKKOR 60 mm f/2.8D lens (Tokyo, Japan). Mounting different bandpass filters on the camera lens enabled VIS and UVF (X-Nite CC1 and B + W 415), NIR (Heliopan RG1000) and UVR photography (Andrea “U” MK II) [22,23,24]. The lighting conditions for VIS and NIR photography involved two 500 W halogen lamps, while the UVF and UVR used two lamps equipped with eight 40 W 365 nm UV fluorescence tubes. The X-Rite ColorChecker Passport (Grand Rapids, MI, USA) was used for the camera calibration and profiling in Adobe Photoshop CC. The images were taken with American Institute of Conservation Photo Documentation (AIC PhD) target to facilitate further processing of RAW files [22]. The infrared false-colour (IRFC) images were obtained by combining VIS and NIR photographs with Adobe Photoshop CC, following the protocol of the American Institute of Conservation [25].

#### 2.2.2. Digital Microscopy

Keyence VHX-6000 (Osaka, Japan) was used for the digital microscopy of the paintings. The instrument was equipped with a zoom lens operating at magnifications of 20×–200×. The images were processed using an integrated Keyence software—VHX-H2M2 and VHX-H4M.

#### 2.2.3. XRR

Radiographic imaging of the paintings was conducted using a Siemens Ysio Max digital X-ray system (Munich, Germany) with a 7 Mpx detector of dimensions 35 × 43 cm. The images were captured at 40 kV, 0.5–2 mAs, 4 s acquisition time and 100 cm distance between the X-ray source and detector. Post-processing work was performed using iQ-LITE and Adobe Photoshop CC 2017 software (San Jose, CA, USA).

#### 2.2.4. Preparation of Samples

The pigments scrapings for the PLM analyses were dispersed on glass microscope slides, mounted with Meltmount (n_D_ = 1.662) from Cargille (Cedar Grove, NJ, USA), which was introduced under the cover glass. Samples intended for the cross-section analyses were embedded within acrylic resin—ClaroCit from Struers (Cleveland, OH, USA). The resin-cast cross sections were ground and polished wet on SiC Foils from Struers down to grade 4000 using grinder-polisher MetaServ 250 from Buehler (Lake Bluff, IL, USA). The samples of fibres were boiled in water and then mounted on glass microscope slides with a drop of water introduced under the cover glass.

#### 2.2.5. OM and PLM

The samples were examined through a Leica DMRX polarised microscope (Wetzlar, Germany) at magnifications of 100×–400×. Transmitted VIS light was used for the PLM observations and fibres morphology, whereas reflected VIS and UV lights were used for the studies of the cross-sections. The samples were photographed using a Leica DFC295 digital camera and further processed with a dedicated software—Leica Application Suite 4.8. The PLM was carried out using the workflow developed by Peter and Ann Mactaggart [26].

#### 2.2.6. Staining Tests

The phloroglucinol stain determined the presence and concentration of lignin in the natural fibres [27,28]. The principle of the test is based on the agent sorbed only by the lignin part of the fibre. Colour reactions were observed in reflected light at magnification of 100×.

#### 2.2.7. FE-SEM-EDS

The paint cross-sections were affixed to the stubs with carbon tapes and examined with FE-SEM Hitachi SU5000 (Tokyo, Japan) coupled with Bruker XFlash^®^ 6/60 EDS (Billerica, MA, USA). The SEM, backscattered electron (BSE) imaging and EDS analyses were carried out using an acceleration voltage of 20 kV, 60 Pa chamber pressure, 50–60 intensity spot, 180 s acquisition time and at 10 mm working distance. The data were acquired and processed using Bruker ESPIRIT 2.0 software.

#### 2.2.8. ATR-FTIR

Analyses were conducted on paint cross-sections with a Bruker Hyperion 3000 FTIR microscope equipped with a mid-band mercury cadmium telluride (MCT) detector coupled with a Vertex 80 FTIR spectrometer. The ATR objective (20×) equipped with a germanium crystal was used for the compression of the samples. The background was measured with 64 scans before each acquisition. Spectra of each sample were recorded over the spectral range 4000 to 600 cm^−1^, with a resolution of 4 cm^−1^, and obtained as a sum of 64 scans. Data were processed and interpreted using Bruker Opus 7.5 software. Additionally, the spectra were interpreted by comparison with references in the material collection of the Institute for Conservation, Restoration and Study of Cultural Heritage, Nicolaus Copernicus University, a spectral library of the Infrared and Raman Users Group (IRUG) [29], as well as Database of ATR-FT-IR spectra of various materials [30].

## 3. Results and Discussion

### 3.1. Characteristics of the Painting Supports

Of the 11 Huangshan and Guilin landscapes, 4 were painted on the unbranded textiles. Based on their structure analyses they are distinguished into three matching types.

**Figure 3:**
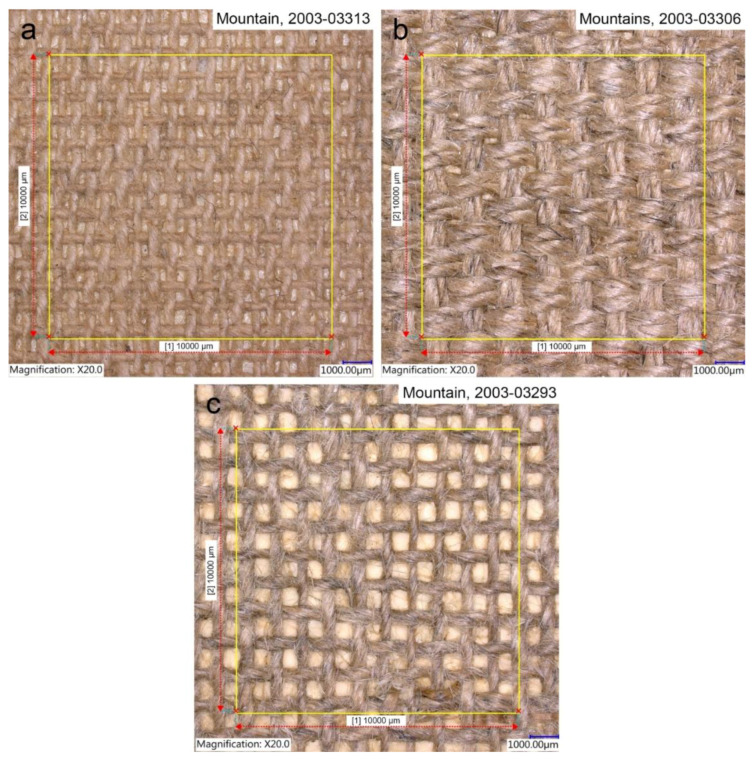
Details of the photomicrographs of three types of canvases identified in the investigated paintings: (**a**) type 1; (**b**) type 2; (**c**) type 3.

**Figure 4:**
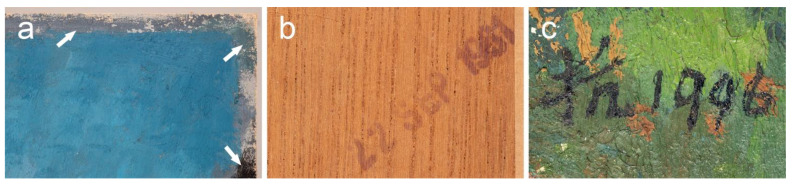
Details of *Mount Huangshan*, 1996, showing the: (**a**) underlying paint scheme indicated by the arrows; (**b**) date stamp on the back of the plywood auxiliary support; (**c**) artist’s signature “Kang” and date “1996” on the painting.

Type 1 was identified in *Mountain* (1981) and *Mount Huangshan* (1996). This type relates to the plain-weave canvases made of S-twisted warp threads and Z-twisted weft threads. Cotton was confirmed by flattened and twisted fibres. The canvases have high density, characterised by a thread count of 17 × 12 per cm (Figure 3a). The intriguing use of these two textiles by Liu Kang, 15 years apart, could be explained by bringing into play his old stock in 1996. However, the initial discovery of another paint scheme underneath *Mount Huangshan* (1996) and the local framer’s date stamp on the back of the plywood auxiliary support strongly suggest that the earlier composition was mounted on the plywood board and framed on 22 September 1981. Then, in 1996 as indicated by the painted date, the artist reused that earlier painting for a new composition (Figure 4).

Types 2 and 3 comprise linen canvases made of Z-twisted weft and warp threads. Linen fibres were identified by their morphological features, such as pronounced dislocations, transverse markings and uneven pink stains, obtained by the phloroglucinol test [27]. However, the discriminating feature of these canvases is the type of weave and density. Hence, the canvas of type 2 is characterised by a basket-weave structure with a thread count of 8 × 10 per cm. It was determined in *Mountains* (1991) (Figure 3b). Type 3 relates to a plain-weave canvas with a thread count of 10 × 10 per cm, and it was identified in *Mountain* (1995) (Figure 3c).

Three investigated paintings are stretched over the unbranded strainers, whereas *Mount Huangshan* (1996) is mounted on the plywood. The visual analyses of the paintings enabled us to distinguish between three types of fastening of the canvas to the auxiliary supports. Regularly distributed nails were observed in the tacking margins of *Mountain* (1981), suggesting commercial stretching of the painting support. However, unevenly spaced staples used for fastening *Mountains* (1991) and *Mountain* (1995), followed by crudely cut edges of the tacking margins, seem to point to the artist’s stretching practice.

**Figure 5:**
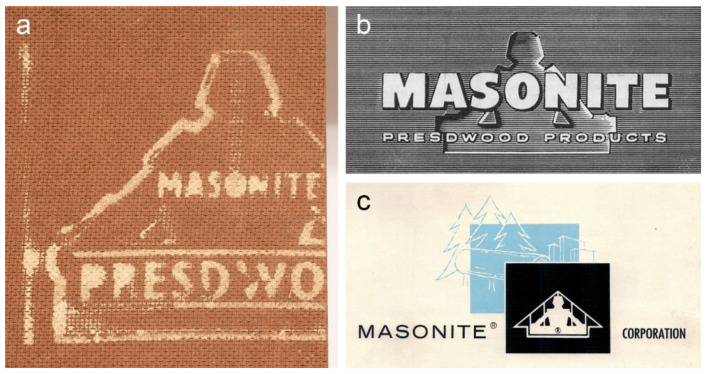
Detail showing the Masonite^®^ Presdwood^®^ stamp on the reverse side of *Mount Huangshan*, 1983 (**a**). Masonite^®^ Presdwood^®^ brand sign featuring the “Masonite Man” from the (**b**) 1950 and (**c**) 1960 Masonite Corporation advertisements.

A sparing utilisation of the canvas supports suggests the artist’s awareness of their limitations in that they would not endure his new way of handling of the paint with heavy impastos and robust scraping. Therefore, the preferential use of 4 mm thick hardboards resembling Masonite^®^ (pressure-moulded wood fibres) is evident in seven paintings. A partially preserved stamp on the reverse side of *Mount Huangshan* (1983) conforms with the Masonite^®^ Presdwood^®^ trade name featuring the “Masonite Man”, which was regularly marketed in the company’s advertisements (Figure 5) [31]. Presdwood^®^ is a hardboard that is smooth on one side and textured on the other due to the screen used to form the fibres in the manufacturing process [32,33]. The hardboards were convenient to use as they did not require a lengthy preparation, compared to the raw canvas. They were light and easy to carry around—an important advantage when painting outdoors. The artist used the smooth side of the boards for painting.

Regarding the source of supply of the painting supports, it remains uncertain whether the artist acquired them in Singapore or in China. The technical data of the painting supports are presented in Table 2.

### 3.2. Characteristics of the Grounds

**Figure 6:**
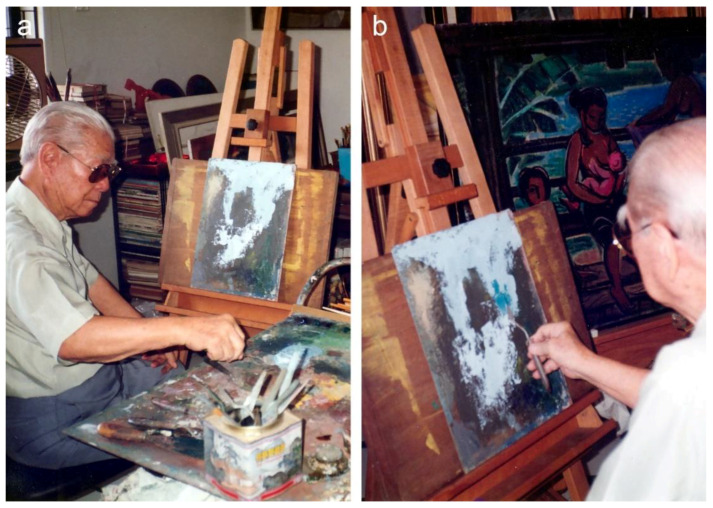
Archival photographs of Liu Kang from the 1990s showing the artist at work. The photographs show the initial underpaint of the sky area conducted over an earlier composition (**a**) and subsequent application of colour for the depiction of vegetation or distant hilltops (**b**). Liu Kang family collection. Images courtesy of the Liu family.

The microscopic analyses revealed that seven paintings on the hardboards were executed without priming. This observation accords with two archival photographs from the 1990s, providing a rare glimpse into the artist’s approach to painting on the hardboard. They document the artist’s initial painting process over a recycled composition on the hardboard without the application of an intermediate ground layer (Figure 6). Nevertheless, the features of the grounds are discussed based on the remaining four canvas paintings.

**Figure 7:**
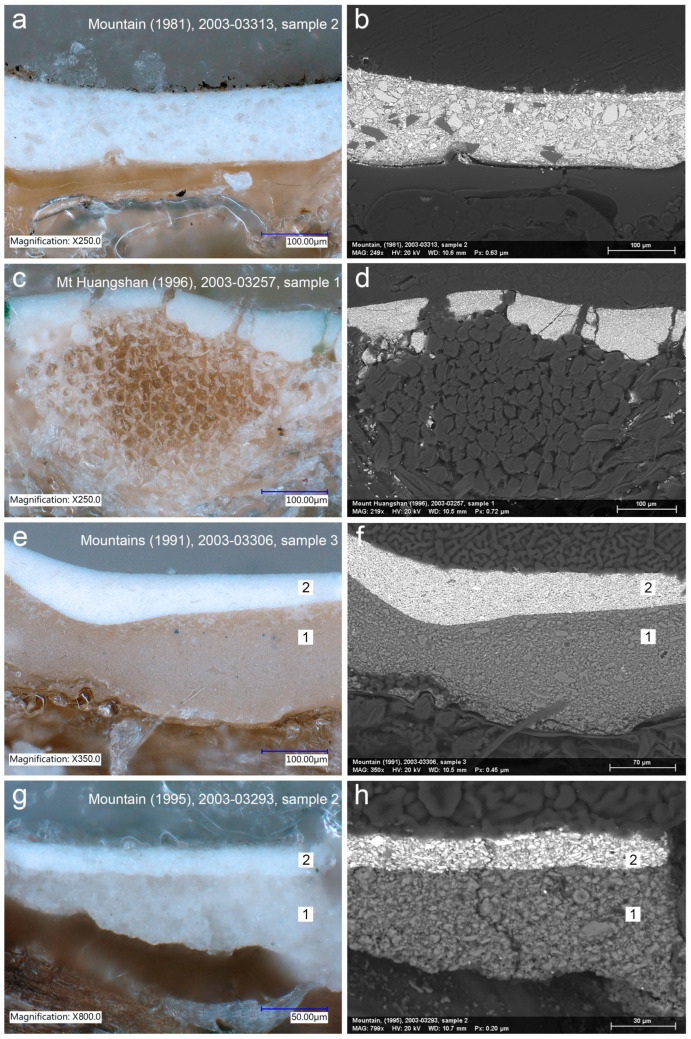
Optical microscopy and corresponding backscattered electron (BSE) images of cross-sections representing two types of identified grounds: (**a**–**d**) single-layered ground of type 1 with coarsely (**b**) and finely (**d**) ground barium white compound particles; (**e**,**g**) double-layered ground of type 2 characterised by a high concentration of chalk in the bottom layer (dark grey) and predominant presence of lead white in the top layer (white) (**f**,**h**). Numbers differentiate between layers of the ground.

Two types of ground preparation were determined. The ground of type 1 relates to the cotton canvas paintings *Mountain* (1981) and *Mount Huangshan* (1996). The microscopic observation revealed that it is a white and single-layered ground. A concomitant presence of Ba, S and Zn elements combined with IR absorption bands at 1066, 983, 630 and 605 cm^−1^ suggested that the ground is primarily composed of lithopone (PW5) and/or barium white (PW21) and zinc white (PW4) [30] with the addition of lead white (PW1) identified by Pb signal and characteristic IR absorption bands at 3528, 1398 and 681 cm^−1^ [30,34]. Interestingly, the SEM-BSE images showed that barium white compound particles are coarsely ground in *Mountain* (1981), while in *Mount Huangshan* (1996), they are in the form of fine grains (Figure 7a–d). Hence, these variations could relate to marginal changes in formulation of the ground or different batches of the ingredients. The presence of drying oil was detected by IR absorption peaks at 2918, 2849, 1736, 1456, 1161 and 721 cm^−1^ [30]. The commercial preparation of the canvas for *Mountain* (1981) is assumed based on the presence of the ground layer on its tacking margins. The evident correlation of commercial preparation and commercial stretching of the canvas indicates that the painting support was bought ready primed on the strainer. Regarding the *Mount Huangshan* (1996), which has tacking margins cut off, the canvas structure and ground preparation share distinctive features with *Mountain* (1981), pointing to the same manufacturer.

Double grounds of the same structure were identified on linen canvases of *Mountains* (1991) and *Mountain* (1995) (Figure 7e–h). Thick bottom layers are composed predominantly of roughly ground chalk particles (PW18) identified by strong C- and Ca-elements and FTIR peaks at, respectively, 1397, 871 and 712 cm^−1^ [30]. Chalk was admixed with lithopone and/or barium white and zinc white, lead white and titanium white (PW6). The latter was assumed by the presence of Ti. The upper layers are thinner than the bottom layers. They were made of the same constituents but mixed in different concentration. Lead white features as the main compound. A drying oil as a binder was confirmed in both layers. An overview of the ground characteristics of the paintings is presented in Table 3.

**Figure 8:**
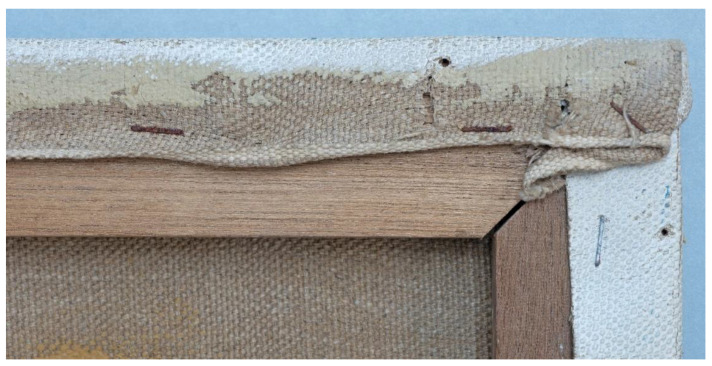
Close-up showing the uneven ground coverage found on the tacking margin of *Mountains*, 1991.

Both *Mountains* (1991) and *Mountain* (1995) show an uneven ground coverage of the tacking margins along one edge (Figure 8). Although considered commercial, this kind of preparation probably resulted from the mounting of long and wide canvas by the unprimed edges on a frame before ground application. This leads to the conclusion that the artist bought such prepared canvases from the roll or by the metre and mounted them over the bare strainers.

### 3.3. Liu Kang’s Paint Brands and Palette of Colours

**Figure 9:**
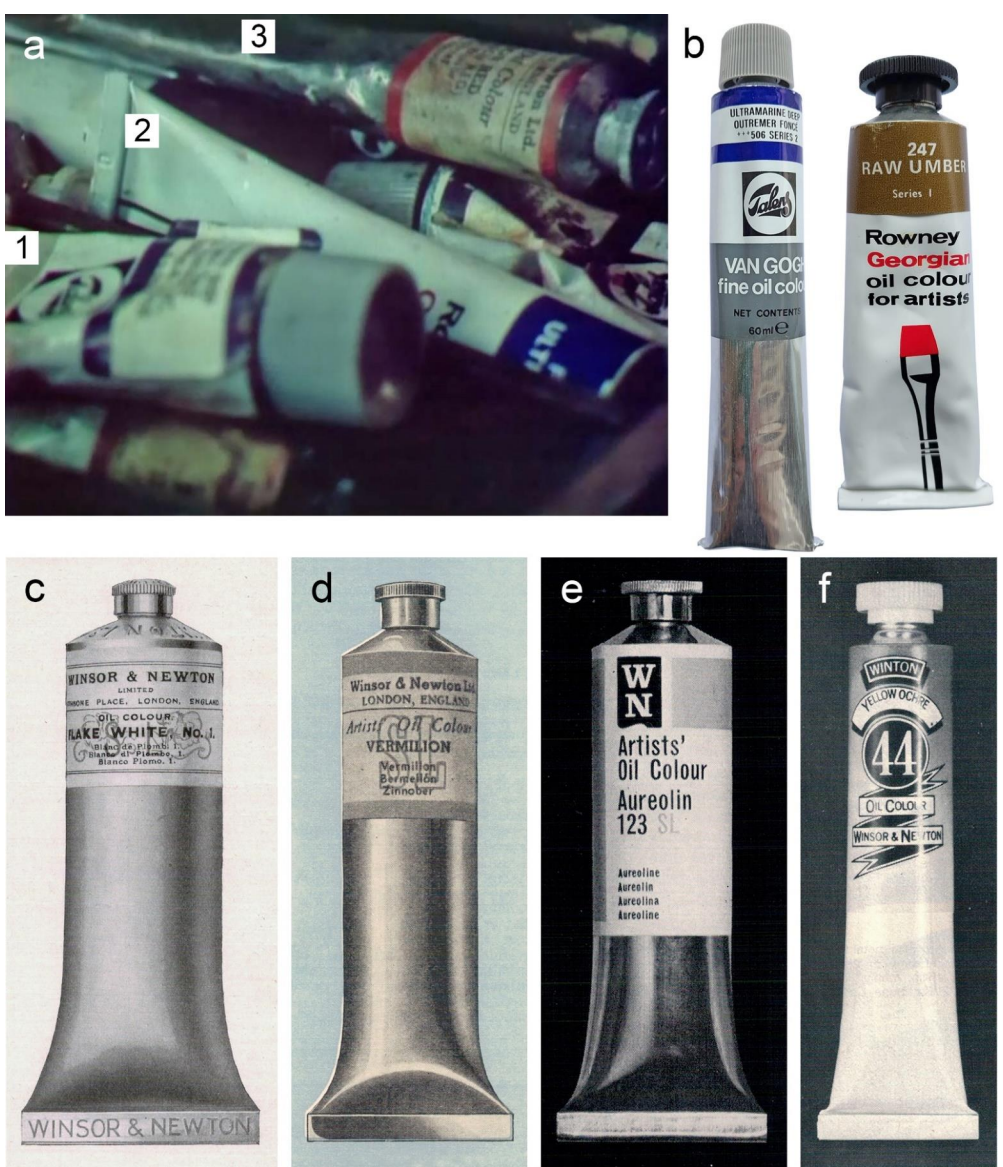
(**a**) Still image from the 1982 TV documentary showing the artist’s paint tubes from Royal Talens (1), Rowney (2) and W&N (3). Copyrights of Mediacorp TV Singapore Pte Ltd. (**b**) Authors reference Royal Talens (Van Gogh series) and Rowney (Georgian series) oil paint tubes from the 1980s. W&N oil paint tubes advertised in the company’s catalogue from: (**c**) 1934, (**d**) 1957, (**e**) 1963 and (**f**) 1979.

To the best of the authors’ knowledge, information about the brand(s) of the paints or pigments used by Liu Kang for the execution of the investigated landscapes is non-existent. As Liu Kang frequently travelled to China, the use of the local Chinese brands can be considered. However, some clues were provided in the TV documentary, *Portrait of an artist: Liu Kang*, presented on 26 February 1982, which featured the artist in his studio using painting materials [35]; when compared against the authors’ references and colourmen catalogues, these materials can be identified as oil paint tubes from Royal Talens (Van Gogh series), Rowney (Georgian series) and Winsor & Newton (W&N) (Figure 9a,b). Although the design and labelling of the Royal Talens and Rowney tubes seen in the documentary relates to those manufactured at the time the documentary was made (Figure 9b), the W&N tube appears to be much older. Based on information available from the W&N catalogues, such as the characteristics of tube design and tube labelling, the authors inferred that similar tubes were in use until at least 1957 (Figure 9c,d). A major change in the design of the tubes was observed in the next available catalogue from 1963 (Figure 9e). The W&N tubes from 1979 also showed a different design (Figure 9f). Nevertheless, as Liu Kang displayed a preference for a bulk purchase of the paint colours [16], the use of the old stock paints like those from W&N is not surprising.

Regarding the artists’ colourmen brand(s) employed by Liu Kang in the 1990s, Royal Talens (Rembrandt series) and Rowney (Georgian series) oil paints were evidenced in the previous research by the authors [16]. Following on this lead, it can be speculated that the investigated landscapes from that period were painted using the same brands of paints. In addition, it is conceivable that, during the painting sessions, the artist mixed the paints of at least three different brands. Hence, the attribution of the identified pigment mixtures to the specific colourmen brand(s) should be made very carefully.

As the subject matter was strongly influenced by fluctuating weather conditions, the painted scenes do not strike the viewer with vivid hues typical for Liu Kang. Instead, the adopted palette of colours is limited; however, the obtained colour mixes prove the artist’s colour sensitivity. The colouristic scheme of the investigated paintings relies on the contrasting juxtaposition of brown hues, mainly characterising the raw rock structures of the foreground and middleground, and blue hues representing distant mountain ranges, sky and water. Green hues of scanty vegetation were the less utilised; however, they play an important role in a visual counterbalancing of the optically heavy foreground mountains. The overview of the pigment analyses is presented in Appendix A, Table A1.

#### 3.3.1. Brown

**Figure 10:**
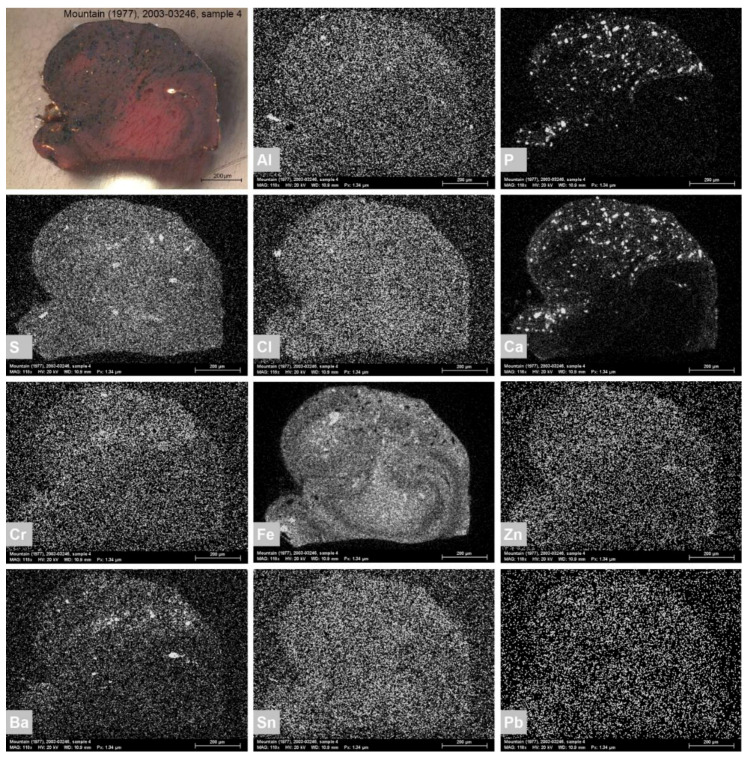
Optical microscopy image of the cross-section of sample 4 at 100× magnification, extracted from *Mountain*, 1977, photographed in VIS (**top-left**), followed by SEM-EDS elemental distribution maps. The greyscale corresponds to the intensity of the signal of each element: white equals high intensity, and black means low intensity. A high intensity of the Fe signal suggests the use of red iron-containing earth. The concomitant presence of Ca and P signals highlights the use of bone black, whereas the co-location of the Al, Cl and Sn elements suggests the presence of organic red on Sn- or Al-based substrate. The concomitant presence of Cr, Zn, Ba and Pb may indicate the use of Cr-containing yellow(s).

**Figure 11:**
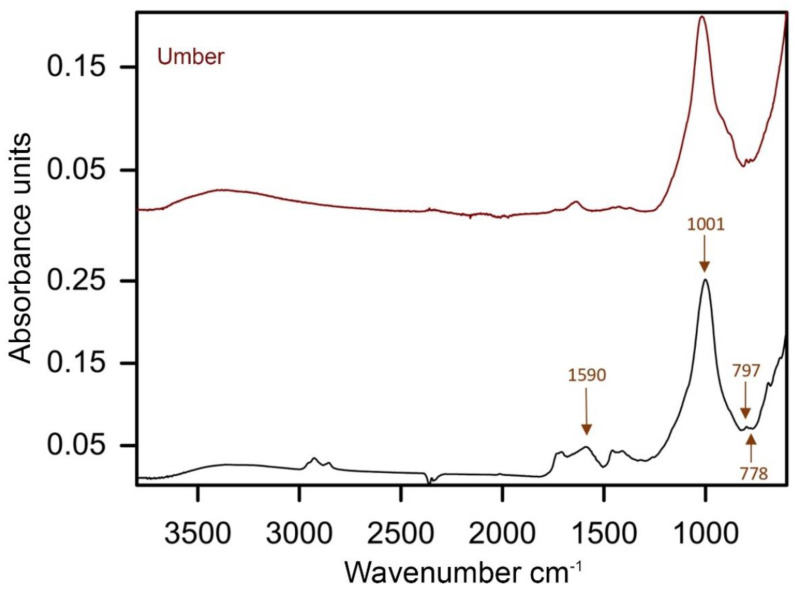
ATR–FTIR spectra of the brown paint of sample 4 extracted from *Mount Huangshan* (1987), with labelled marker peaks of umber and reference spectra of the same pigment.

The analyses indicate that the brown hues are primarily composed of yellow and red iron-containing earths. They were suspected based on the PLM observation of a considerable amount of yellow and red particles with high refractive indexes, followed by SEM-EDS recording of distinct Fe signals and FTIR detection of additive minerals such as kaolinite (absorption peaks at 1030, 1000, 915 and 798 cm^−1^) and gypsum (absorption peaks at 3533, 3401, 1094, 669 and 605 cm^−1^) [30,36]. However, the FTIR confirmation of iron-containing earths was hindered by the overlapping signals of other compounds present in the paint mixtures, whereas the iron oxide IR absorption bands at 530 and 450 cm^−1^ were beyond the spectral range of the instrument. A concomitant presence of Ca and P elements and IR absorption peak at 1023 cm^−1^ were associated with bone black admixture (PBk9) (PO_4_^3−^ stretching) (Figure 10) [37]. The addition of umber (PBr7) was assumed in five brown paint samples based on the concomitant presence of Fe and Mn elements and absorption peaks at 1590, 1001, 797 and 778 cm^−1^ as sample 4 extracted from *Mount Huangshan* (1987) (Figure 11) [30]. The PLM observation of some organic red particles with a unique low refractive index suggested minor admixtures of organic red(s). However, their unequivocal identification was complicated due to a low concentration of the pigment in the investigated brown mixtures. Nevertheless, SEM-EDS of sample 4 from *Mountain* (1977) enabled the detection of trace Cl, Sn and Al signals, suggesting an organic red on Sn- or Al-based substrate (Figure 10) [38,39,40]. Further FTIR analyses of the sample recorded IR absorption peaks at 3063, 3028, 1666, 1592, 1547, 1380, 1286, 955, 915, 866, 753, 693 and 672 cm^−1^, indicative of red azo pigment naphthol red AS-D (PR112). Its admixture was also detected in sample 3 from *Mount Huangshan* (1983) based on the IR absorption peaks matching the reference sample at 1591, 1495, 1316, 1262, 1241, 1118, 1026, 881 and 700 cm^−1^, and the IRUG reference [41]. Moreover, a concomitant presence of Cr, Zn, Ba and Pb allowed us to consider a Cr-containing yellow(s) (Figure 10). However, further PLM and FTIR analyses could not provide a precise identification, except sample 8 from *Mount Huangshan* (1996). IR absorption peaks at 1081, 1028, 853 and 843 cm^−1^ evidenced the presence of chrome yellow (PY34) [30].

A trace presence of other organic reds, probably on an Al-containing substrate, was suspected in two more brown paint samples.

**Figure A1:**
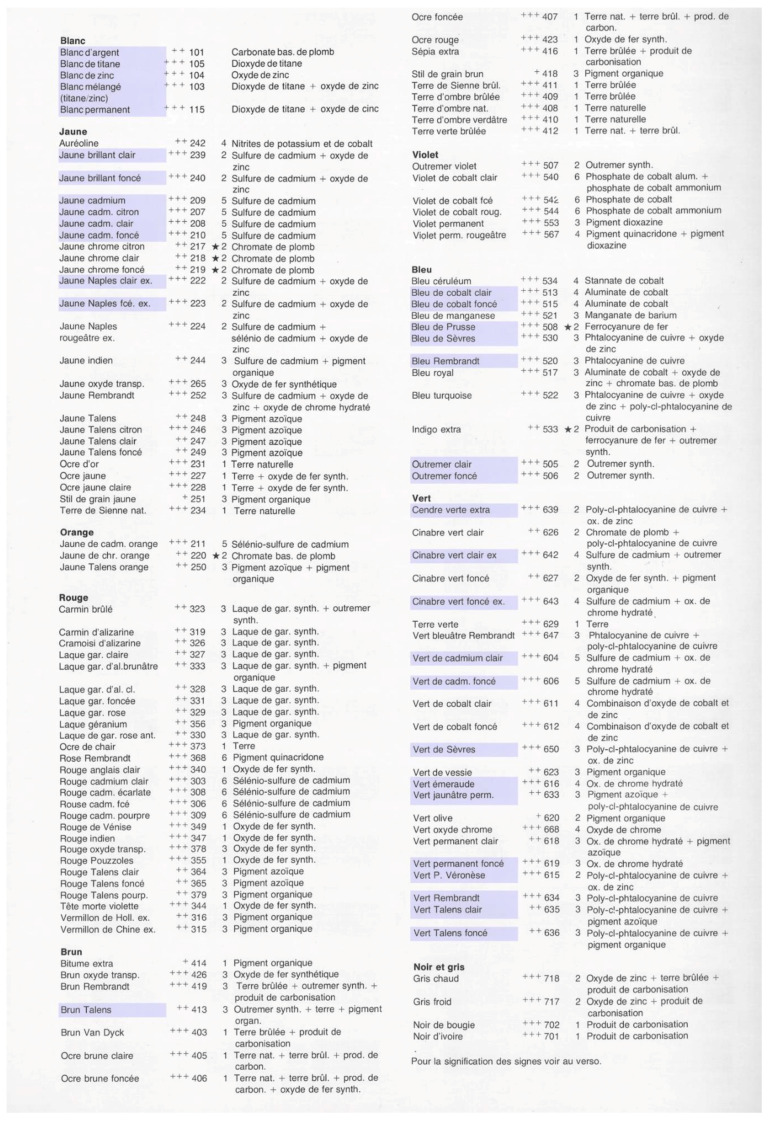
Oil colours and their pigment compositions, listed in the catalogue of Royal Talens (French edition) from 1982. The highlighted details indicate colours that could be tentatively linked to the identified pigment mixtures.

Interestingly, the PLM observation of blue isotropic particles, which turn red with a Chelsea filter and have a low refractive index, suggested the use of ultramarine (PB29) in four analysed brown paints. This outcome corroborated the SEM-EDS detection of Na, Al, Si and S elements. However, FTIR analyses did not permit a conclusive attribution due to the signal interferences of different compounds within similar spectral ranges. Nevertheless, ultramarine could be considered as contamination or a deliberate admixture by the artist to enhance a depth of dark brown brushstrokes. However, according to the 1982 Royal Talens catalogue, this blue was combined with some earth pigment and an unspecified organic pigment, obtaining Talens brown paint (Appendix A, Figure A1). Interestingly, the brown hue of the sample 8 from *Mount Huangshan* (1996) was additionally modified with Prussian blue (PB27). This blue pigment was identified by PLM (observation of dark blue isotropic and low refractive index particles, which turn dark green under a Chelsea filter), SEM-EDS detection of Fe and FTIR absorption peak at 2089 cm^−1^ [30].

#### 3.3.2. Yellow and Orange

**Figure A2:**
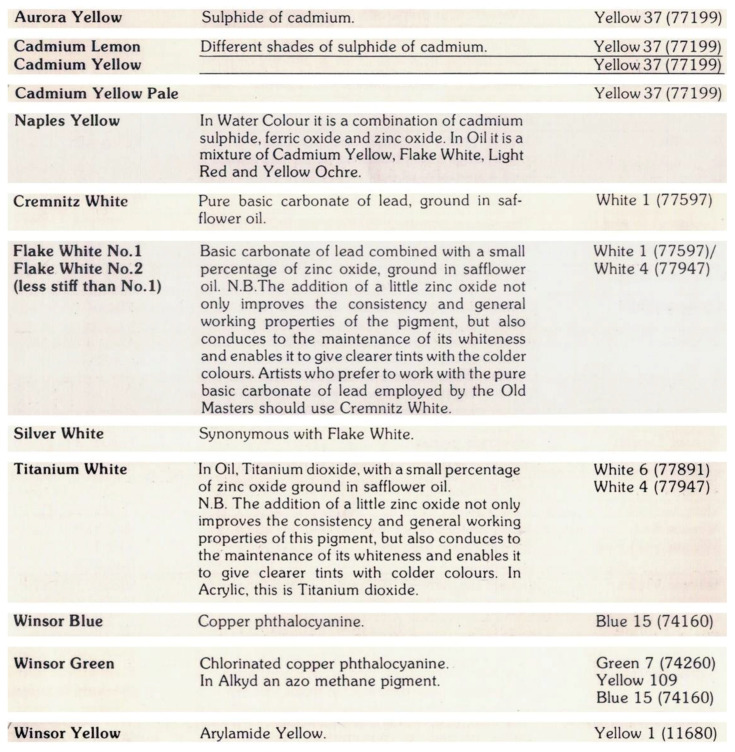
Selected compositions of pigments, listed in the catalogue of W&N from 1982, used for manufacturing of oil, watercolours, acrylic and alkyd paints for artists.

The analyses of the yellow and orange hues indicated a consistent use of yellow iron-containing earths at variable concentrations in combination with different pigments. For instance, the PLM observation of large and rough anisotropic green particles with a high refractive index tentatively suggested the admixture of viridian (PG18) in the paint sample 7 from *Mountain* (1981) [26,42]. The FTIR spectroscopy of the sample confirmed viridian only by 1068 and 801 cm^−1^ absorption bands as the fingerprint region of this pigment (600–400 cm^−1^) is behind the spectral range of the measurement. Moreover, an admixture of yellow organic pigment Hansa yellow G (PY1) was confirmed by IR absorption bands at 1666, 1599, 1561, 1508, 1491, 1344, 1294, 1270, 1236, 1175, 952, 911, 801, 771 and 756 cm^−1^ in the same sample [43,44]. Royal Talens listed four variants of organic azo yellow pigments in the 1982 catalogue; however, the company did not specify their variants. Hansa yellow G was available from W&N under the name Winsor yellow, according to their 1982 catalogue (Appendix A, Figure A1 and Figure A2).

**Figure 12:**
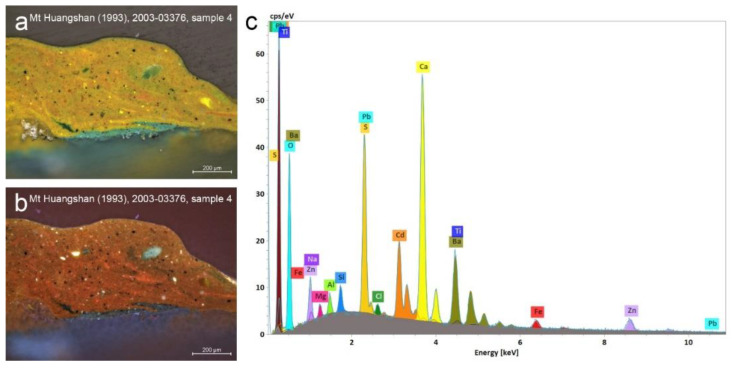
Optical microscopy images of the paint cross-section of sample 4, extracted from *Mount Huangshan*, 1993, photographed in: (**a**) VIS and (**b**) UV. The corresponding SEM-EDS spectra of the yellow paint (**c**). Red fluorescence of yellow paint and strong Cd and S signals combined with Ba and Zn suggests the use of cadmium yellow or cadmopone or zinc-modified light cadmium yellow.

Admixtures of cadmium-containing yellow were initially determined based on the characteristic red UV fluorescence of the yellow particles in sample 4 from *Mount Huangshan* (1993) and sample 7 from *Mount Huangshan* (1996) [22]. This observation was further confirmed by coinciding Cd, S, Ba and Zn signals, suggesting the presence of cadmium yellow (PY35) or cadmopone (co-precipitated cadmium sulfide and barium sulfate) or zinc-modified light cadmium yellow (Figure 12). FTIR did not permit an unequivocal identification of cadmium yellow due to its characteristic bands occurring outside of the spectral range of the instrument [45]. Nevertheless, the obtained data correlate with the materials available from Royal Talens and W&N in 1982. Royal Talens listed four shades of cadmium yellow paint composed of cadmium sulfide and two variants of Naples yellow as well as two variants of bright yellow both composed of cadmium sulfide and zinc oxide. W&N listed four shades of cadmium yellow and their Naples yellow was a mixture of cadmium yellow with other pigments (Appendix A, Figure A1 and Figure A2). FTIR analyses of sample 7 from *Mount Huangshan* (1996) identified naphthol red AS-D validated by the absorption peaks at 3274, 3234, 3185, 3123, 3075, 1667, 1603, 1594, 1545, 1478, 1447, 1365, 1324, 1278, 1257, 1203, 1153, 1120, 1071, 1046, 1012, 964, 901, 891, 872, 748, 697 and 665 cm^−1^. The additional presence of the Sn and Cl elements in sample 7 from *Mount Huangshan* (1996) could point to another organic red on Sn-containing substrate added to the paint mixture to obtain an orange hue. Yellow-green colour brush dabs found in *Mount Huangshan* (1994) are composed of partially mixed yellow iron-rich earth, ultramarine and probably some Co-containing pigment (sample 7), which could be cobalt blue or green; however, its low concentration did not permit PLM observation.

#### 3.3.3. Blue

The analytical results indicate that blue painted areas were achieved through an extensive use of ultramarine admixed frequently with other pigments to obtain the desired hue. Hence, the addition of cobalt blue (PB29) was suspected based on the co-location of Co and Al elements and PLM observation of isotropic particles with a high refractive index that appear red with a Chelsea filter [26,42]. Another blue pigment tentatively identified in the mixtures with ultramarine is Prussian blue. It was assumed based on the Fe signal and PLM observation. [26,42]. Interestingly, a trace Cu signal was recorded in sample 7 from *Mount Huangshan* (1986), suggesting an admixture or contamination with phthalocyanine blue (PB15). This result was further confirmed with FTIR by a complex array of the absorption peaks at 1506, 1416, 1335,1287, 1164, 1118, 1089, 900, 769, 754 and 729 cm^−1^ [46,47]. The analyses of the blue paint samples suggested some admixtures of viridian, yellow or red iron-rich earths, cadmium yellow or its variant and bone black assumed based upon the PLM and SEM-EDS analyses.

Viridian was assumed based on the PLM observation and detection of Cr signal. It was further confirmed by absorbances at 1063 and 796 cm^−1^ as in sample 8 from *Mount Huangshan* (1993) [30]. Phthalocyanine blue was available from Royal Talens in two shades—Rembrandt blue, composed solely of copper phthalocyanine, and Sèvres blue, containing copper phthalocyanine and zinc oxide. This blue pigment was also listed by W&N as Winsor blue (Appendix A, Figure A1 and Figure A2).

#### 3.3.4. Green

**Figure 13:**
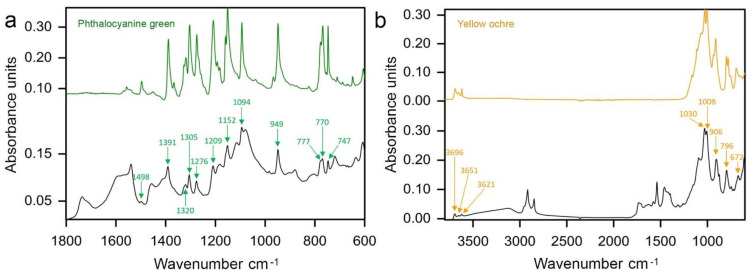
(**a**) ATR–FTIR spectra of the green paint of sample 4 extracted from *Mount Huangshan* (1983), with labelled marker peaks of phthalocyanine green and reference spectra of the same pigment. (**b**) ATR–FTIR spectra of the green paint of sample 4 extracted from *Mount Huangshan* (1986), with labelled marker peaks of yellow ochre admixture and reference spectra of the same pigment.

The evidence collected from the analysed samples showed some diversity of the selected pigments in obtaining green hues by the artist. By far, viridian is the most frequently occurring green pigment in green paint samples. However, it was used mainly in combination with yellow iron-rich earths, some ultramarine, Prussian blue, cobalt blue, cadmium yellow or its variant or zinc yellow. Although the mixture of viridian with yellow iron-rich earths or ultramarine is considered as artist-made, the combination of viridian and cadmium yellow or its variant detected in sample 5 from *Mount Huangshan* (1993) might be related to commercially prepared cadmium green light, cadmium green dark or cinnabar green dark available from Royal Talens (Appendix A, Figure A1). However, a trace Cd signal in the investigated sample may also suggest contamination as cadmium yellow or its variant was the principal component of the yellow brushstroke in the same artwork (sample 4). SEM-EDS detection of Cu and Cl elements in conjunction with FTIR measurements may account for phthalocyanine green (PG7) in green paints from three investigated green paint samples. The most representative spectra of this green pigment with IR signature peaks at 1498, 1391, 1320, 1305, 1276, 1209, 1152, 1094, 949, 777, 770 and 747 cm^−1^ were recorded from sample 4 extracted from *Mount Huangshan* (1983) (Figure 13a) [48]. Phthalocyanine green was available from W&N as a Winsor Green and from Royal Talens as Rembrandt green and was sold in seven variants as a combination with zinc white and/or organic pigments (Appendix A, Figure A1 and Figure A2). Artist made admixture of yellow ochre (PY43) to phthalocyanine green was detected in sample 4 from *Mount Huangshan* (1986). Yellow ochre was confirmed based on the PLM, high concentration of Fe and IR absorption peaks at 3696, 3651, 3621, 1030, 1008, 906, 796 and 672 cm^−1^ (Figure 13b) [30].

**Figure 14:**
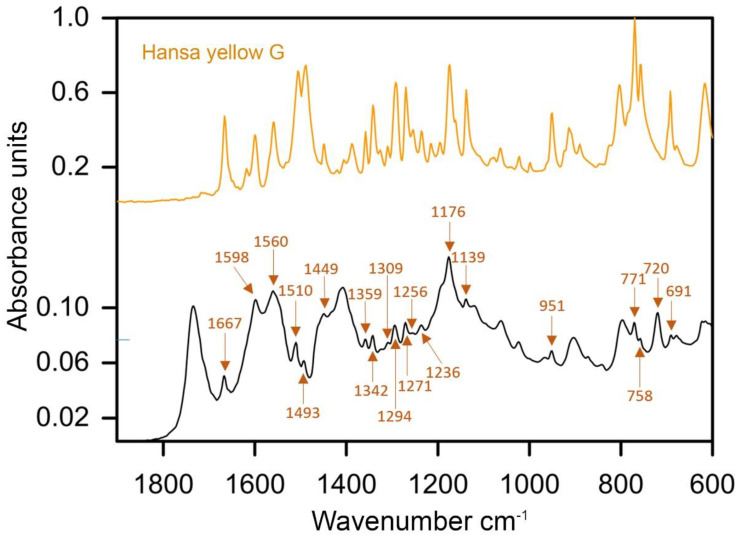
ATR–FTIR spectra of the green paint of sample 3 extracted from *Mount Huangshan* (1995), with labelled marker peaks of Hansa yellow G and reference spectra of the same pigment.

In addition to viridian and phthalocyanine green, the artist frequently combined ultramarine with yellow iron-rich earths and Hansa yellow G. FTIR analyses of the sample 3 from *Mount Huangshan* (1995) revealed the most representative peaks of Hansa yellow G at 1667, 1598, 1560, 1510, 1493, 1449, 1359, 1342, 1309, 1294, 1271, 1256, 1236, 1176, 1139, 951, 771, 758, 720 and 691 cm^−1^ (Figure 14) [43]. A mixture of ultramarine with cadmium yellow or its variant was assumed in the sample 3 from *Mountain* (1981). A homogenous structure of the paint layer observed on the cross-section may be associated with a commercial mixture, and such was listed in the 1982 Royal Talens catalogue under the name of cinnabar green light (Appendix A, Figure A1).

**Figure 15:**
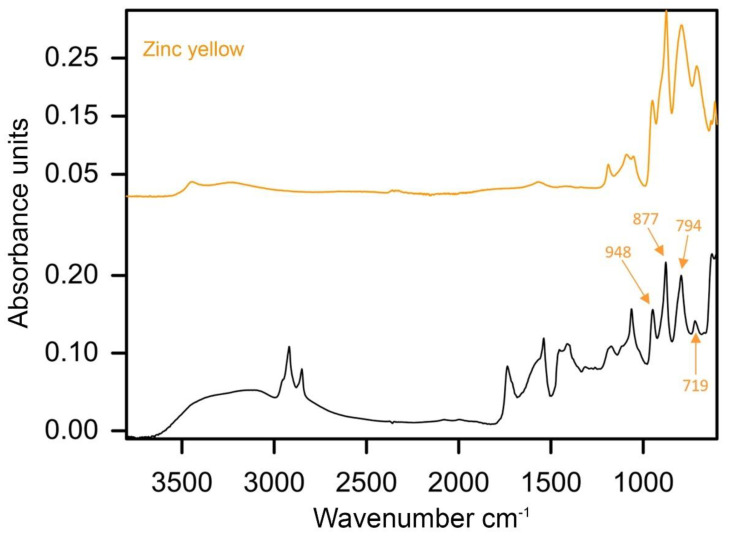
ATR–FTIR spectra of the green paint of sample 1 extracted from *Mountain* (1977), with labelled marker peaks of zinc yellow admixture and reference spectra of the same pigment.

The role of Prussian blue in the development of green hues was generally considered marginal, as it was detected only in three green paint samples. For instance, it was used as a minute admixture to viridian and zinc yellow as in sample 1 from *Mountain* (1977). Although the FTIR measurement did not permit the detection of this blue pigment due to its low concentration, some characteristic blue particles were observed in PLM and SEM-EDS detected trace Fe signal. This result is compliant with the IRFC imaging of the sampling area, as the dark-violet colour is determined by the purple representation of viridian, combined with a dark-blue representation of Prussian blue. Although very little of Prussian blue was added to the paint, this outcome is possible due to a high tinting strength achieved with at low concentration of the pigment [49]. The zinc yellow (PY36) admixture was confirmed with FTIR by absorption peaks at 948, 877, 794 and 719 cm^−1^ (Figure 15) [50].

The analyses of sample 4 from *Mount Huangshan* (1983) revealed that Prussian blue combined with phthalocyanine green was the main constituent of some brushstrokes employed for the depiction of the foreground greenery. Prussian blue was assumed by PLM, SEM-EDS and FTIR by the peak at 2091 cm^−1^, relating to C≡N stretching [51]. This outcome is consistent with the IRFC imaging, as blue representation of Prussian blue combined with purple representation of phthalocyanine green can produce violet.

**Figure 16:**
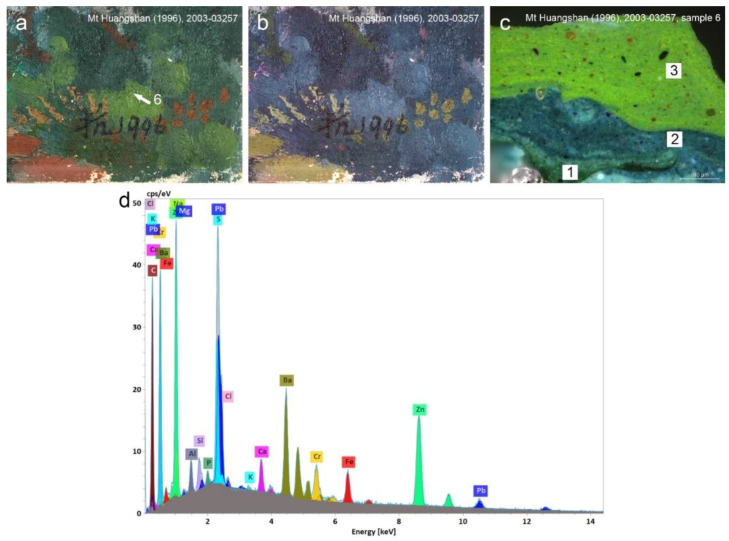
Detail of *Mount Huangshan*, 1996, photographed in VIS showing the extraction spot for sample 6 (**a**) and IRFC image of the same area (**b**). Optical microscopy image of the cross-section of the sample 6 at 200× magnification. Numbers indicate different layers of the paint structure (**c**). Corresponding SEM-EDS spectra of the green paint from layer 3 of sample 6 extracted from the sampling spot (**d**). A green colour recorded as blue in IRFC and the high concentration of Fe in the layer 3 suggested a presence of Prussian blue, which when combined with chrome yellow (assumed by strong Cr and Pb signals), resulted in the green hue of the layer.

The admixture of Prussian blue was also confirmed by IR absorption peak at 2088 cm^−1^ in sample 6 from *Mount Huangshan* (1996). The pigment was combined with chrome yellow. Chrome yellow was positively identified by combining strong Cr and Pb signals and some characteristic FTIR bands at 1049, 855, 831 and 626 cm^−1^ [30]. A high concentration of Zn, Ba and S may indicate a barium white extender of chrome yellow and the admixture of zinc white or lithopone [52] (Figure 16).

Interestingly, the analyses of paint sample 3 from *Mount Huangshan* (1994) revealed a trace concentration of Co which, linked with Al or Zn, also present in the paint layer, may account for Co-containing pigments such as cobalt green or cobalt blue. Unfortunately, the PLM observation was not conclusive, probably due to the low concentration of the pigments in question in the sample, whereas FTIR provided only an absorption band at 640 cm^−1^, which was insufficient to unequivocally determine the presence of cobalt blue or cobalt green [53]. However, a coinciding use of cobalt blue for developing a sky of the same composition (sample 2) may suggest the contamination of the green paint mixture with this blue pigment. The bone black found in a few green paint samples was probably employed as a minor admixture to modify green hues.

#### 3.3.5. White and Black

**Figure A3:**
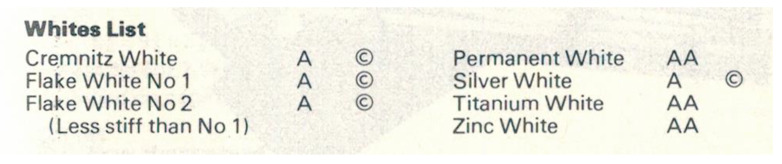
White oil colours listed in the catalogue of W&N from 1979.

Although the artist did not incorporate pure white highlights, white paints combined with some blue, brown, green and black pigments were extensively used to depict clouds. Thus, titanium white appears to be the primary white pigment, used not only in the white painted areas but also in other parts of the compositions as an admixture to produce the desired tints. Moreover, all investigated paint mixtures have in common the notable presence of lithopone and/or barium white and zinc white. However, an unequivocal determination of whether these compounds relate to titanium white as extenders or other incorporated pigments is complicated. Nevertheless, titanium white was available from Royal Talens in 1982 as pure titanium dioxide and in combination with zinc white under the name of mixed white and permanent white. W&N admixed titanium white with some zinc white according to their 1982 catalogue (Appendix A, Figure A1 and Figure A2). The additional presence of Ca recorded in the majority of white paint mixtures could be attributed to chalk, yellow iron-rich earth admixtures [36] and bone black. Although at a low concentration, lead white was detected in three white paint samples. This white pigment also frequently appears in combination with other pigments, but its brightening role seems to be limited by the concurrent and prevailing presence of titanium white. Although progressively restricted from the market, lead white was still available from Royal Talens in 1982. Pure lead white was also listed in the W&N catalogues from 1979 and 1982 under the name of cremnitz white, whereas flake white and silver white usually contained an admixture of zinc white (Appendix A, Figure A1, Figure A2 and Figure A3). Regarding other pigments present in the white mixtures, ultramarine appears consistently, and it is very often paired with yellow iron-rich earths, probably to obtain greenish hues. The PLM and SEM-EDS analyses suggested that viridian and cobalt blue occurred intermittently at low concentrations in white mixtures.

Liu Kang did not employ pure black brushstrokes during the execution of the investigated landscapes. However, a trace presence of Ca and P elements and FTIR peak at 1023 cm^−1^ suggested a minor addition of bone black to modify the hue of complex paint mixtures.

### 3.4. Binding Media and Other Identified Compounds

FTIR analyses of all paint samples confirmed the use of a drying oil as a binding medium (Appendix A, Table A1). The spectra showed typical IR absorption bands at 2923, 2853, 1737, 1460, 1235, 1160, 1098 and 721 cm^−1^. Additionally, thanks to the typical IR absorption band at 1540 cm^−1^, a frequent occurrence of zinc soaps was detected. This result is consistent with the observation of zinc soaps in Liu Kang’s nude paintings from 1992 to 1999 and probably painted with similar brands of the materials. The zinc soaps may be present in the investigated paint layers as a result of the reaction between the metal ions of the zinc-containing pigments and free fatty acids [54,55]. However, conservation problems, such as cleavage, paint loss, disfiguring lumps, increased transparency and surface efflorescence, associated with zinc soaps have not been observed in the investigated paintings.

### 3.5. Sketches, Drawings, Photographs and Preparatory Underdrawings

**Figure 17:**
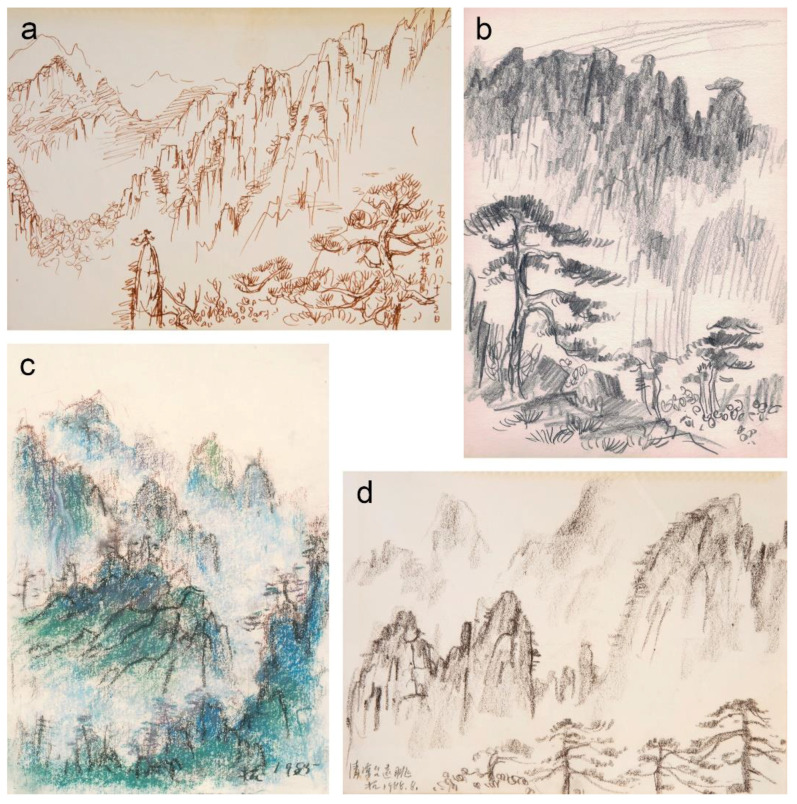
(**a**) Liu Kang, *Mount Huangshan*, 1988, pen on paper, 35 × 25.5 cm; (**b**) Liu Kang, *Mount Huangshan*, 1970s, pencil on paper, 18 × 25 cm; (**c**) Liu Kang, *Mount Huangshan*, 1985, pastel on paper, 35 × 25 cm; (**d**) Liu Kang, *Mount Huangshan*, 1988, charcoal on paper, 35 × 25.5 cm. Images (**a**,**d**) are from Liu Kang family collection. Images courtesy of Liu family. Images (**b**,**c**) are gifts of the artist’s family. Collection of National Gallery Singapore.

Previous research about Liu Kang revealed that he was a prolific sketcher whose complex drawing studies of the subject matter preceded the transference of the settled ideas onto the canvas [6,16,56]. Hence, in preparation for the painting of the Huangshan and Guilin landscapes, his approach did not differ from his earlier established practice and it resulted in an impressive number of pen, pencil, pastel and charcoal sketches and drawings, which form an important part of Liu Kang’s artistic output (Figure 17).

**Figure 18:**
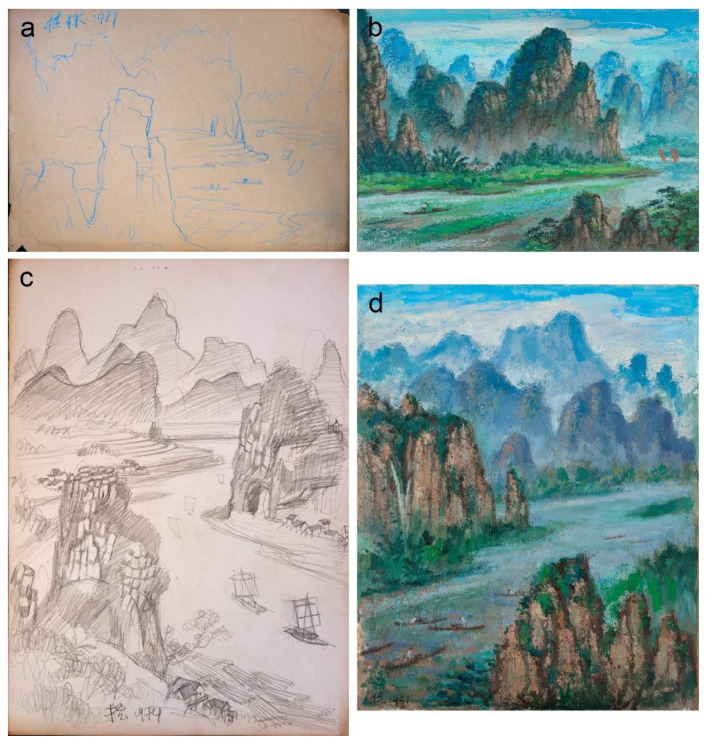
(**a**) Liu Kang, untitled, 1979, crayon on paper, 38 × 28 cm; (**b**) Liu Kang, *Mountain*, 1995, oil on canvas, 84.7 × 118.5 cm; (**c**) Liu Kang, untitled, 1979, pencil on paper, 38 × 28 cm; (**d**) Liu Kang, *Mountains*, 1991, oil on canvas, 76.5 × 61 cm. Images (**a**,**c**) are from Liu Kang family collection. Images courtesy of Liu family. Images (**b**,**d**) are gifts of the artist’s family. Collection of National Gallery Singapore.

**Figure 19:**
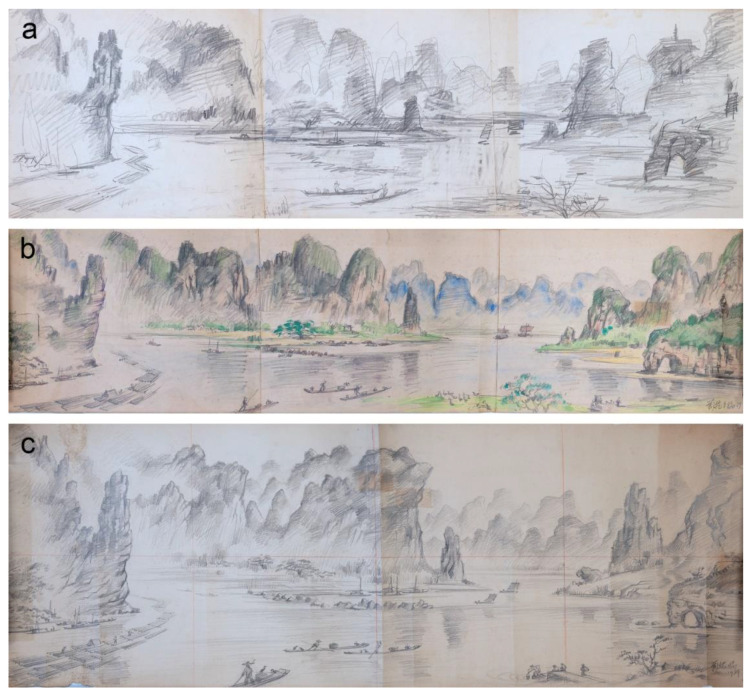
(**a**) Liu Kang, untitled, undated, pencil on paper, 26 × 88 cm; (**b**) Liu Kang, untitled, 1979, pencil and watercolour on paper, 28 × 107 cm; (**c**) Liu Kang, untitled, 1979, pencil on paper, 38 × 107 cm. Liu Kang family collection. Images courtesy of Liu family.

Most of these sketches were executed rapidly, suggesting an initial probing of the subject matter to search for its potential as a painting composition. However, the comparative studies with the investigated paintings did not reveal direct similarities in the compositional details. Nevertheless, one view of Guilin mountains particularly attracted the artist’s attention, resulting in a remarkable series of sketches and drawings from 1979, suggesting preparation for a major undertaking. Liu Kang probably began with two drawings that could have determined the idea for further detailed studies on paper and canvas. A view of the mountain peaks and river bend with boats in the focal point depicted in the blue crayon drawing from 1979 appears to be similar to that from *Mountain* (1995), probably recorded from the higher vantage point (Figure 18a,b). The drawing was executed with several free-flowing lines without shading. The other pencil drawing also from 1979 is technically advanced and contains more details. Its vertical orientation with composition constructed on strong divisions between the visual planes resembles *Mountains* (1991) (Figure 18c,d). Both drawings have the isolated rock formation emphasising the foreground, while the middleground encompassed the boat traffic in the mountain river with characterised in detail one mountain, and background composed of two mountain ranges with obliterated details due to aerial perspective. Despite these compositional similarities, the drawing and *Mountains* (1991) depict views from different observation points. The subject of the mountain river evolved further into three panoramic drawings that show the progression of details controlled by advanced shading and the introduction of colour in water-based technique (Figure 19). Unfortunately, the panoramic drawings and paintings—*Mountains* (1991) and *Mountain* (1995)—do not share any distinctive landscape features that could unequivocally link them together.

**Figure 20:**
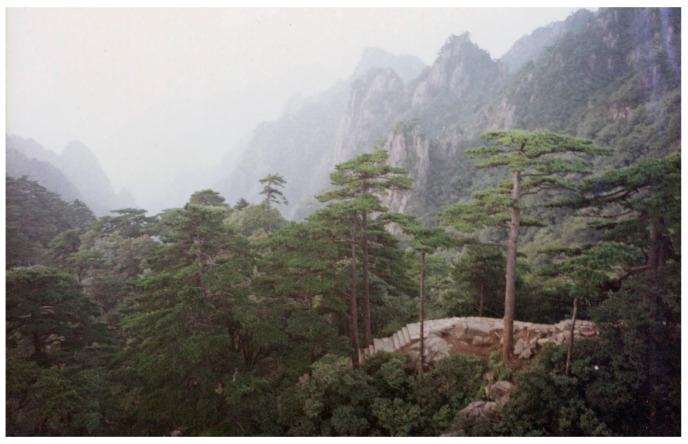
Archival, undated photograph of Huangshan mountains by Liu Kang. Liu Kang family collection. Image courtesy of Liu family.

Liu Kang produced numerous undated photographs of Huangshan and Guilin landscapes that accompanied his sketches and drawings. They record a good deal of details and how they disappear in the misty mountain air (Figure 20). The camera was a very practical tool for the artist, and it is known that he used it extensively for recording subjects that interested him, treating the device like a sketchbook [6,56,57]. However, the available photographs did not show any correlation with the investigated paintings. Nevertheless, the variety of techniques employed by the artist to capture the scenic views suggest that they could have served as a source of inspiration for the creation of the compositions executed on the canvas. Moreover, considering that the development of the colour film rolls and images in the 1980s and 1990s required several days, the photographs were likely taken to document the interesting views for future reference than to use in the actual location.

Judging by the extensive collection of Liu Kang’s sketches, drawings and photographs of Huangshan and Guilin landscapes, one would expect the presence of elaborate preparatory drawings in the investigated paintings. Unfortunately, VIS and NIR examinations did not provide evidence of the underdrawings or discernible painterly contours. This could be due to the artist’s painting technique, which efficiently conceals any preparatory layers, or an intentional omission of that phase in the creative process, the latter as illustrated in the photographs of Liu Kang at work taken in the 1990s (Figure 6a,b). The photographs capture an early stage of his painting process, most likely of some mountains, conducted over an earlier composition. Nevertheless, it is noteworthy that no compositional lines are visible, suggesting that he painted from memory or based his painting on a drawing or photograph that was sufficient for settling the composition. However, more technical studies of these paintings would be required to confirm these suggestions.

### 3.6. Development of Paintings

There is currently little information regarding Liu Kang’s approach to painting the Huangshan and Guilin landscapes. The artist had never talked about the nuances of his working practice; very little archival material documenting him at work has been preserved; and the paintings have not been the subject of in-depth studies. However, thanks to a photographs of the artist in his studio taken in the 1990s, we can see him at an early stage of the painted composition, which could have been a mountainous landscape (Figure 6a,b). The initial painting steps were focused on establishing the structure of the composition and its dominant colours. He achieved that fairly simply with a palette knife and a decisive and broad application of a bright underpaint for the depiction of clouds or sky (Figure 6a). The subsequent application of blue-green colour patches, using the same tool, suggests that he started the layering process to characterise vegetation or distant hilltops (Figure 6b). A close look at his container with tools revealed that the artist intended to execute the painting only with palette knives (Figure 6a). Although the broad underpaint of the composition appears to be typical for the artist’s initial approach to painting [6,13], further execution of the Huangshan and Guilin landscapes stands out from his earlier genres and artistic phases mainly due to the use of a limited palette of colours and a consistent shift towards rich texture as a way of artistic expression.

Despite his predilection for a solid build-up of paint layers in his earlier oeuvre, his abundant use of paint and its creative manipulation for achieving a repertoire of artistic effects sets a new stylistic quality in the investigated paintings.

**Figure 21:**
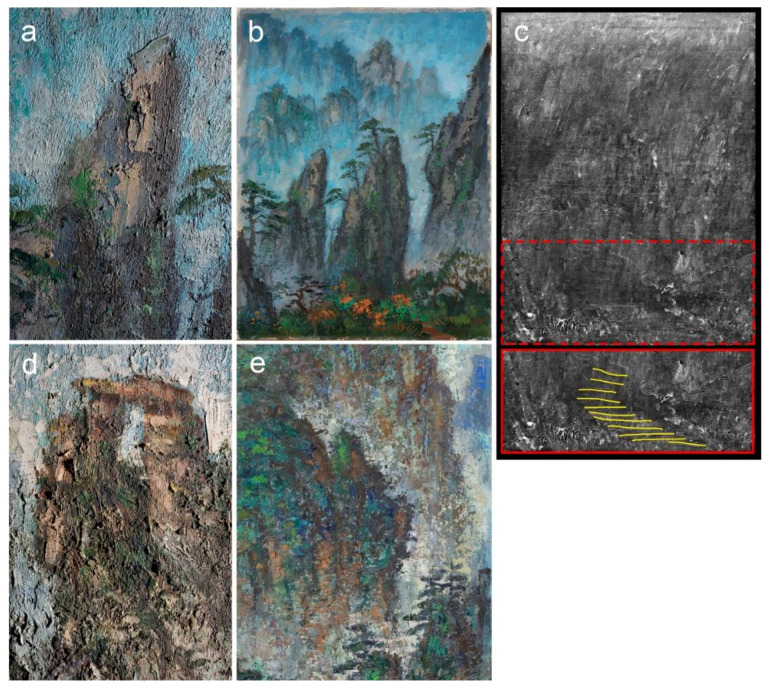
(**a**) Detail of *Mount Huangshan*, 1996, showing a brush and palette knife paint application. (**b**) Image of the same painting photographed in VIS and (**c**) corresponding XRR image and detail with superimposed tracing of the winding path. (**d**) Detail of *Mountain*, 1981, showing high impastos. (**e**) Detail of *Mount Huangshan*, 1986, showing the juxtaposing touches of paint for the execution of the misty and translucent air.

Both *Mountain* (1977) and *Mount Huangshan* (1996) share a conventional handling of the paint. They were executed without hesitation wet-on-wet in dynamic brushstrokes (Figure 1a and Figure 2e). Despite these similarities, some high impastos appear in *Mount Huangshan* (1996). They were introduced with palette knives and brushes within the foreground mountain peaks and foliage of the vegetation (Figure 21a). Such an approach could have been a result of the artist reworking of an earlier composition from 1981, as indicated in this article based on the canvas and ground analyses. The comparison of VIS and XRR images of the painting revealed that the underlying composition could be a vertically oriented mountainous winding path—seen in the foreground (Figure 21b,c). Hence, it could be speculated that the artist’s idea for repainting a former artwork in 1996 was confined to major changes to the foreground areas, some minor shifts in the middle ground and a new colour of the background mountain ranges. Therefore, it is possible that he did not see a need for an extensive use of a palette knife to underpaint a large area for the new composition. Instead, most of the work was executed with brushes, while a palette knife was used for some minor impastos.

Although the overall impression of the paint texture in *Mountain* (1977) and *Mount Huangshan* (1996) is of a moderate level, the subsequent artworks from 1981 to 1995 demonstrate further development of the paint application methods toward the impasted and complex layer build-up to emphasise the subject matter. Hence, the manipulation of generously applied paint is achieved by the alternate use of brush and palette knife in both wet-on-wet and wet-on-dry colour patches. In particular, the varied brush sizes enabled multidirectional and tight dabbing, resulting in an accumulation of heavy impastos that emphasises the sculptural quality of the objects and isolates these objects from the surrounding environment. This approach turned out to be very successful in the characterisation of the rough texture of the rock masses and vegetation in *Mountain* (1981), *Mount Huangshan* (1983) and *Mount Huangshan* (1993) (Figure 21d). It is also apparent from all investigated paintings that, typical for Liu Kang, continuous and bold outlines of the shapes gave way to the complex texture to enhance forms emerging from the clouds. However, some reduced contours were incorporated for a depiction of the shadows and some foreground structures.

Clouds and mist played an important role in rendering the volatility of weather conditions, aerial perspective and separation of the landscape planes. These features were painted more softly than mountains and rock structures. The opaque clouds were obtained with a grey paint deftly spread with the palette knife, as can be exemplified in *Mount Huangshan* (1994) (Figure 2b). The misty air was rendered with heavily brightened blue and green tints with varied size brushstrokes juxtaposed with underlying colours relating to the mountains or blue sky. The best example of such execution can be observed in *Mount Huangshan* (1983) and *Mount Huangshan* (1986) (Figure 21e).

Regarding the vegetation, the artist frequently used small brushes for a calligraphic depiction of the trunks and limbs of trees, whereas the foliage was depicted using brushes and palette knives paint touches. It is also apparent from all the investigated paintings that the bold outlines of the shapes, Liu Kang is known for, were used intermittently and with reduced intensity as modulated texture sufficiently enhanced the forms.

**Figure 22:**
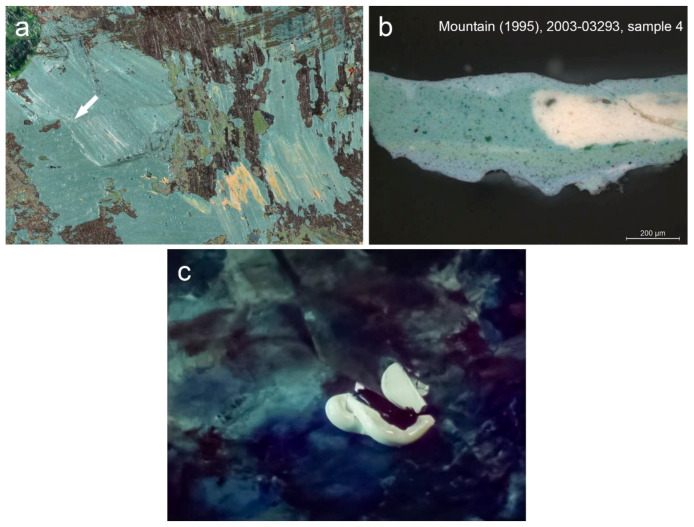
(**a**) Detail of partially mixed paint in *Mountain*, 1995. The arrow indicates the sampling spot. (**b**) Microscopy image of the cross-section of sample 4 taken from the sampling spot. (**c**) Still image from the 1982 TV documentary evidencing that the artist’s paint mixing technique resulted in a partially mixed paint application. Copyrights of Mediacorp TV Singapore Pte Ltd.

Due to the complex paint application technique, the artworks were likely executed in more than one sitting. A disadvantage of this approach is a low sense of freshness and spontaneity observed in *Mount Huangshan* (1986), *Mount Huangshan* (1987) and *Mountain* (1995) (Figure 1d,e and Figure 2d). Rendering of the compositional elements is monotonous mainly because of small dabs of paint and poor light effects. Nevertheless, the remaining paintings evoke the immediacy of the shape of the mountains in an expressive way. An interesting aspect of his technique is the occasionally occurring multicolour paint touches (Figure 22a) resulting from tools that are not completely cleaned, or from partially mixed paint. These features were additionally evidenced in the paint cross-sections, which show poorly blended raw colours within a single layer (Figure 22b). Moreover, this painting approach was recorded in a 1982 TV documentary about the artist (Figure 22c), revealing a cursory mixing of blue and white paints with a palette knife [35].

### 3.7. Artist’s Practice of Reusing Earlier Paintings

**Figure 23:**
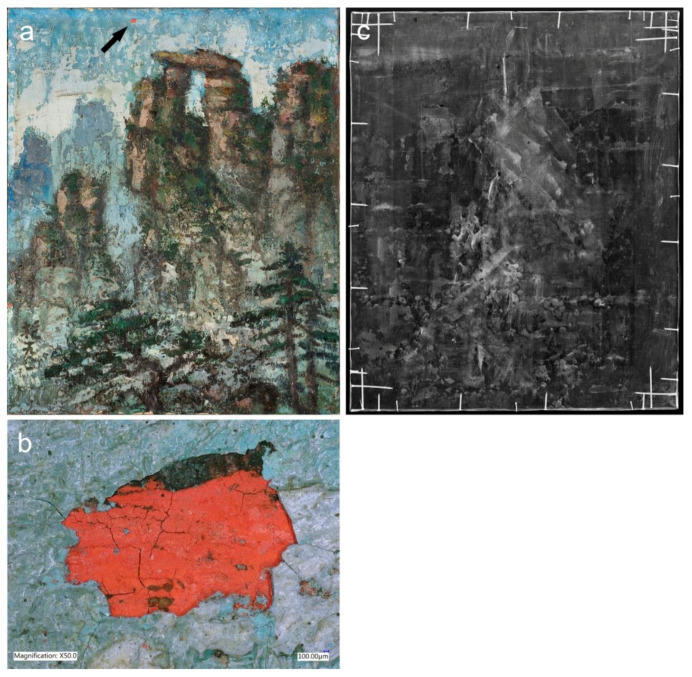
(**a**) Image of *Mountain*, 1981, photographed in VIS with the marked location of paint loss revealing a different paint scheme beneath the current composition. (**b**) Digital microscope image of red and black paint layer discernible through the paint loss of the same painting. (**c**) XRR image of the painting showing a poor rendering of the underlying composition.

Combined use of the surface digital microscopy, VIS raking light imaging and XRR suggested that nine paintings underwent major alterations or were created over the earlier artworks. The evidence of the underlying paint schemes was found along the edges of the paintings (Figure 4a), through the losses of the current compositions or based on the unusual paint texture and XRR (Figure 23). However, the visualisation of hidden paintings was hampered by the complex paint application system of the current compositions. For this reason, VIS raking light photography revealed only the most pronounced paint texture relating to the hidden brushwork. The radiographic images mostly recorded the variations in the thickness of the paint layers as the primary source of contrast, resulting in poor rendering of the underlying compositions [58]. The thickness of the paint layers of the final images also significantly reduced the NIR penetrative capability. Nevertheless, some compositional details of the rejected composition were recorded with XRR in the painting *Mount Huangshan* (1996) (Figure 21c). In addition to the above-mentioned fragmentary evidence of the underlying paint schemes, Liu Kang’s painting process over the recycled composition was documented in two archival photographs from the 1990s (Figure 6). These discoveries are especially interesting as they suggest that utilising unsatisfactory paintings became a norm during the twilight of Liu Kang’s professional career.

### 3.8. Provenance of the Paintings

Of particular interest is establishing the provenance of the artworks. A preferential use of hardboards by Liu Kang appears to be adequate for outdoor painting. However, judging from the analytical data and archival photographs, it is fair to say that studio work played an important role in the development of the paintings—from the initial underpaint to final touches—which were often carried out over the recycled artworks in more than a single session (Figure 6). These findings converge with Liu Kang’s 1981 interview, revealing: “He is now working on a series of landscapes on the theme of Huang-shan, the fabled mountains for Chinese landscape painters” [59]. Considering that the interview was conducted with the artist in Singapore, he likely worked concurrently on the mentioned landscapes in the studio. Moreover, due to his deteriorating eyesight in 1980s and 1990s, his painting productivity was reduced. In 1989 interview, he revealed: “I can’t paint for periods now. Before, I could paint through the whole day. Now, I get tired. It is a problem” [60]. In 1992, the eyesight in his left eye was significantly reduced. However, the cornea transplant in 1993 enabled him to regain full functionality. Liu Kang made a reference to his laborious painting practice caused by worsening eyesight in the 1993 interview: “Since I couldn’t see with my left eye, I am painting at much slower pace now. A painting which needed only two to three days to do in the past now takes me about two to three weeks” [61]. Judging from his health problems, it is very unlikely that the artist painted outdoor as this type of painting approach is characterised by rapid execution, usually in one or two sittings. Moreover, the detected combination of wet-on-wet and wet-on-dry paint application system reinforces this notion. Therefore, it seems reasonable to conclude that Liu Kang’s main objective during his trips to China was forming an extensive visual library for future reference, whereas paintings were executed in his studio in Singapore based on photographs and sketches. Hence, his choice of hardboards was determined by the material’s ability to endure his vigorous and complex way of handling of the paint contrary to the canvas.

## 4. Conclusions

The complementary use of analytical techniques, archival sources and art historical research significantly advanced our understanding of Liu Kang’s painting materials and the working process employed for the creation of the Huangshan and Guilin landscapes.

As the investigated paintings span the period from 1977 to 1996, one would expect that the artist’s painting methods and materials evolved during that time. Interestingly, this study showed otherwise. A degree of consistency with minor variability in his choice of materials and working practice was observed.

The technical examination highlighted a preferential use of hardboards resembling Masonite^®^ Presdwood^®^. This type of the painting support was probably determined by the dynamic handling of the paint with the palette knives and brushes. The artist chose to paint on the hardboards directly, without a layer of the primer. Commercially made, oil-based grounds were found on a total of four cotton and linen canvases. The structure of the grounds strongly correlates with the type of the canvas. Hence, a single-layered mixture of lithopone and/or barium white and zinc white with some lead white was applied on dense cotton canvases. Linen canvases, although of different weave and density, were primed with double-layered grounds characterised by similar chemical compositions. The thick bottom layers comprise chalk mixed with lithopone and/or barium white and zinc white, lead white and titanium white. The upper layers are thinner than the bottom, but they are made of the same constituents, albeit mixed in different concentrations.

This study did not identify any preparatory underdrawings on the painting supports. The reason could be reduced NIR penetration capability through the thick paint layers or the artist’s deliberate decision to skip the underdrawing stage and confidently establish the compositions with a broad laying of the colours. His painting technique is characterised by a skilled combination of wet-on-wet and wet-on-dry paint application systems with the alternate use of brushes and palette knives, resulting in peculiar impastos. Hence, the development of the compositions was probably laborious and involved more than one sitting. Moreover, the formation of an extensive reference material for future use and the evident practice of recycling earlier artworks suggests studio work rather than plein air sessions in China.

Given the fact that he used Royal Talens, Rowney and W&N oil paints in 1982 and the 1990s, it seems reasonable to conclude that the investigated landscapes were more likely executed using the same brands of the paints. The analytical results show that the artist employed a limited palette of colours characterised by a prevailing use of ultramarine, yellow and red iron-containing earths, viridian and titanium white. Other identified pigments used intermittently or at low concentrations are Prussian blue, cobalt blue, phthalocyanine blue, phthalocyanine green, naphthol red AS-D, umber, Cr-containing yellow(s), cadmium yellow or its variant(s), Hansa yellow G, lithopone and/or barium white and zinc white and bone black

Overall, the artist’s predilection for a conventional and consistent colour scheme for depicting his subject matter is discernible. The artist mixed the paints on the palette rather than used them straight from the tubes. However, some identified pigment combinations suggest commercial mixtures, for instance, cadmium yellow with viridian or ultramarine and titanium white with zinc white. This interpretation was rendered based on the cross-referencing of the analytical data with Royal Talens’s and W&N descriptions of the chemical composition of their oil paints in 1982 catalogues, which are contemporary to some of the landscapes created by the artist. Nevertheless, the information provided in the catalogues may be considered insufficient. Therefore, the analytical studies engaging reference samples of Royal Talens, Rowney and W&N would provide unambiguous information.

In addition to providing salient technical information regarding Liu Kang’s painting process and materials, this study contributes to the growing knowledge of modern painting materials. The collected information may be taken as a reference for further in-depth research aiming to validate the use of Royal Talens, Rowney and W&N oil colours by the artist in the 1980s and 1990s.

## Figures and Tables

**Table 1:** Inventory and technical information of the paintings by Liu Kang included in this study.

Title and Inventory Number	Date	Dimensions H × W (cm)	Primary Support	Auxiliary Support
*Mountain*, 2003-03246	1977	38 × 45.5	Board	
*Mountain*, 2003-03313	1981	36 × 29.5	Canvas	Strainer
*Mount Huangshan*, 2003-03304	1983	84.6 × 64.5	Board	
*Mount Huangshan*, 2003-03327	1986	74 × 48.5	Board	
*Mount Huangshan*, 2003-03251	1987	71 × 55.6	Board	
*Mountains*, 2003-03306	1991	76.5 × 61	Canvas	Strainer
*Mount Huangshan*, 2003-03376	1993	40.5 × 31.7	Board	
*Mount Huangshan*, 2003-03307	1994	78.5 × 58.3	Board	
*Mount Huangshan*, 2003-03378	1995	30.5 × 40.5	Board	
*Mountain*, 2003-03293	1995	84.7 × 118.5	Canvas	Strainer
*Mount Huangshan*, 2003-03257	1996	64 × 49	Canvas	Board

**Table 2:** Overview of the canvas characteristics of the investigated paintings.

Title and Inventory Number	Date	Average Thread Count/cm	Weave	Direction of Warp	Twist	Fibre	Weave Matching Group
*Mountain*, 2003-03313	1981	17 × 12	Plain	Vertical	S (warp) Z (weft)	Cotton	1
*Mountains*, 2003-03306	1991	8 × 10	Basket	Horizontal	Z	Linen	2
*Mountain*, 2003-03293	1995	10 × 10	Plain	Vertical	Z	Linen	3
*Mount Huangshan*, 2003-03257	1996	17 × 12	Plain	Vertical	S (warp) Z (weft)	Cotton	1

**Table 3:** Overview of the ground characteristics of the investigated paintings.

Title and Inventory Number	Date	Sample, Layer Number	SEM-EDS Detected Elements *	FTIR Identification	Result	Type of Ground
*Mountain*, 2003-03313	1981	2, 1	**Ba, O, C**, Zn, Pb, S, Si, (Na, Sr, Al)	Lithopone and/or barium white and zinc white, lead white, zinc soap, oil	Lithopone and/or barium white and zinc white, lead white	1
*Mountains*, 2003-03306	1991	3, 2	**C, O, Pb, Ba**, Ti, Zn, S, Ca, (Na, Si, Al)	Chalk, lithopone and/or barium white and zinc white, oil	Lead white, lithopone and/or barium white and zinc white, titanium white, chalk	2
		3, 1	**O, C, Ca**, Zn, (Pb, Na, Si, Ba, Ti, S, Al)	Chalk, zinc soap, oil	Chalk, lithopone and/or barium white and zinc white, lead white, titanium white	
*Mountain*, 2003-03293	1995	2, 2	**C, O, Ti, Pb, Ba**, Zn, S, Ca, Na, (Al, Si, Cl)	Lithopone and/or barium white and zinc white, lead white, oil	Titanium white, lead white, lithopone and/or barium white and zinc white, chalk	2
		2, 1	**O, Ca, C**, Zn, Pb, (Ba, Na, Ti, Si, Al, S, Cl)	Chalk, lithopone and/or barium white and zinc white, oil	Chalk, lithopone and/or barium white and zinc white, lead white, titanium white	
*Mount Huangshan*, 2003-03257	1996	1, 1	**C, O, Zn, Ba**, S, Na, (Si, Cl, Al, P, Pb)	Lithopone and/or barium white and zinc white, zinc soap, oil	Lithopone and/or barium white and zinc white, lead white	1

* Major elements are provided in bold type, minor elements in plain type and trace elements in brackets.

## Data Availability

The data presented in this study are available upon request from the corresponding author.

## Appendix A

**Table A1:** Summary of the materials identified or tentatively determined in the paint samples extracted from the investigated paintings.

Title and Inventory Number	Date	Colour	Sample	SEM-EDS * Detected Elements	PLM, SEM-EDS Tentative Assignments	FTIR Identification
*Mountain*, 2003-03246	1977	Brown	3	**C, O**, Ca, Zn, Ti, Ba, S, Fe, Na, (Si, P, Al, Pb, Mg, Cl, Sr)	Lithopone and/or barium white and zinc white, titanium white, red iron-rich earth pigment, bone black, lead white	
		Brown	4	**C, O, Fe**, Si, Ca, S, (Cl, Pb, Sr, P, Ba, Na, Zn, Cr, Sn, Al, Ti)	Red iron-rich earth pigment, bone black, lithopone and/or barium white and zinc white, Cr-containing yellow(s), organic red on Sn and/or Al-containing substrate, titanium white	Lithopone and/or barium white and zinc white, iron-rich earth pigment, bone black, naphthol red AS-D, oil
		Blue	5	**C, O**, Na, Al, Si, S, Ca, (Sr, Zn, Mg, K, Ti, Fe)	Ultramarine, chalk, zinc white, titanium white	
		Blue	7	**C, O**, Ca, Zn, Ti, Ba, S, Na, (Al, P, Mg, Fe, Si)	Lithopone and/or barium white and zinc white, titanium white, ultramarine, bone black	
		Blue	9	**C, O**, Ca, Zn, Ti, Ba, S, Na, (Al, Pb, Mg, Si, Cl)	Chalk, lithopone and/or barium white and zinc white, titanium white, ultramarine, lead white	
		Green	1	**C, O, Cr**, Zn, Ba, K, Na, (S, Al, Ca, Mg, Si, Ti, Cl, Fe, P)	Viridian, lithopone and/or barium white and zinc white, bone black, titanium white, Prussian blue	Viridian, lithopone and/or barium white and zinc white, zinc yellow, zinc soap, oil
		White	8	**C, O**, Ca, Zn, Ti, Ba, S, Na, (Mg, Si, Al, P)	Lithopone and/or barium white and zinc white, titanium white, ultramarine, bone black	
*Mountain*, 2003-03313	1981	Brown	8	**C, O, Ba**, S, Al, Zn, Na, Sr, Si, Fe, (Pb, Ca, P)	Lithopone and/or barium white and zinc white, ultramarine, yellow iron-rich earth pigment, lead white, bone black	Lithopone and/or barium white and zinc white, bone black, zinc soap, oil
		Yellow	7	**O, C, Ba**, Zn, Ti, Ca, Cr, S, Fe, Si, Al, Na, (Mg, P, Pb, K)	Lithopone and/or barium white and zinc white, titanium white, chalk, viridian, yellow iron-rich earth pigment, bone black	Lithopone and/or barium white and zinc white, chalk, Hansa Yellow G, viridian, yellow iron-rich earth pigment, zinc soap, oil
		Blue	4	**C, O, Zn**, Ca, Ba, Ti, S, Pb, Na, Al, (Si, Mg, Cd, Cl, Fe, P, Sr, Cr)	Lithopone and/or barium white and zinc white, titanium white, lead white, ultramarine, cadmium yellow or its variant, bone black	
		Blue	6	**Zn, C, O, Na**, (Al, Si, Ba, S, Ti, Ca)	Lithopone and/or barium white and zinc white, ultramarine, titanium white, chalk	
		Green	3	**C, O, Zn**, Ca, Ba, Cd, Na, S, (Cl, Si, Mg, Al)	Lithopone and/or barium white and zinc white, chalk, cadmium yellow or its variant, ultramarine	Lithopone and/or barium white and zinc white, chalk, zinc soap, oil
		Green	9	**C, O**, Pb, Ba, Zn, Al, S, Cr, Si, Fe, (Na, Ti, Ca, Cl, Mg)	Lead white, lithopone and/or barium white and zinc white, ultramarine, viridian, chalk	
		White	5	**C, O, Ba**, Ti, S, Zn, Al, (Na, Si, Ca, Sr, P)	Lithopone and/or barium white and zinc white, titanium white, ultramarine, bone black	
*Mount Huangshan*, 2003-03304	1983	Brown	3	**Zn, C, O, Na**, Fe, Ba, S, (Cr, Al, Si, K, Pb, Ti)	Lithopone and/or barium white and zinc white, red iron-rich earth pigment, Cr-containing yellow, organic red, lead white, titanium white	Lithopone and/or barium white and zinc white, naphthol red AS-D, zinc soap, oil
		Brown	6	**Zn, C, O**, Na, Cr, Ba, K, (S, Al, Fe, Si, Pb, Cl, Ti)	Lithopone and/or barium white and zinc white, Cr-containing yellow(s), ultramarine, red iron-rich earth pigment, titanium white	
		Blue	2	**C, O, Ba**, Ca, Zn, S, Al, Na, Ti, (Pb, Si, Mg, Cl)	Lithopone and/or barium white and zinc white, chalk, ultramarine, titanium white, lead white	
		Green	4	**Zn, C, O**, Ba, Cl, Na, Al, S, Si, (Cr, Fe, Mg, Cu, K, Pb, Sr, Ca)	Lithopone and/or barium white and zinc white, phthalocyanine green, Prussian blue, lead white, chalk	Lithopone and/or barium white and zinc white, phthalocyanine green, Prussian blue, zinc soap, oil
		Green	5	**C, O, Zn, Ba**, Al, S, Na, Cl, Ti, (Si, Ca, Pb, Sr, Mg, Fe)	Lithopone and/or barium white and zinc white, ultramarine, titanium white, chalk, lead white	Lithopone and/or barium white and zinc white, phthalocyanine green, ultramarine, zinc soap, oil
		White	1	**Zn, C, O**, Na, Ti, Ca, (Ba, Fe, Al, Si, S)	Lithopone and/or barium white and zinc white, titanium white, chalk, ultramarine, yellow iron-rich earth pigment	
*Mount Huangshan*, 2003-03327	1986	Brown	5	**C, O, Zn**, Fe, Ti, Ca, Na, Mg, Si, Al, (P, Sr, S, Mn)	Zinc white, red iron-rich earth pigment, titanium white, organic red on Al-containing substrate, umber, bone black	Red iron-rich earth pigment, organic red, chalk, zinc soap, oil
		Blue	2	**C, Zn, O**, Na, Al, S, Ba, Ti, Si, (Ca, Fe, Cl, K)	Lithopone and/or barium white and zinc white, ultramarine, titanium white, chalk, Prussian blue	
		Blue	7	**C, O**, Al, Zn, Co, Ca, Si, Na, (Mg, S, Fe, Cu, Ti, Cl, Pb, Cr, P)	Ultramarine, zinc white, cobalt blue, phthalocyanine blue, titanium white, viridian, lead white, bone black	Phthalocyanine blue, cobalt blue, chalk, zinc soap, oil
		Green	3	**C, O, Zn**, Ca, Na, Ba, S, Si, Fe, (Al, Cl, Mg, Ti)	Lithopone and/or barium white and zinc white, chalk, ultramarine, yellow iron-rich earth pigment, titanium white	
		Green	4	**C, O, Fe**, Ca, Si, Al, Ba, S, Cl, (Ti, Zn, Mg, Cu)	Yellow iron-rich earth pigment, chalk, lithopone and/or barium white and zinc white, titanium white, phthalocyanine green	Yellow ochre, lithopone and/or barium white and zinc white, chalk, phthalocyanine green, zinc soap, oil
		White	6	**C, O, Ti**, Zn, Ca, Mg, Al, Na, (Fe, S, Si, Cl)	Titanium white, zinc white, chalk, ultramarine, yellow iron-rich earth pigment	
*Mount Huangshan*, 2003-03251	1987	Brown	4	**C, O, Fe**, Al, Si, Na, S, Ca, Ba, (P, Ti, Mn, Zn, Mg, Pb, K, Cl)	Umber, bone black, lithopone and/or barium white and zinc white, titanium white, lead white	Umber, lithopone and/or barium white and zinc white, oil
		Blue	2	**C, O**, Ti, Zn, Ca, Mg, Al, Na, (S, Si, Fe, Cl)	Titanium white, zinc white, chalk, ultramarine, Prussian blue	
		Blue	5	**O, Zn, Ba**, C, S, Na, Al, Ti, Si, Ca, (Mg, Fe, Cl, Sr, K, Pb)	Lithopone and/or barium white and zinc white, titanium white, ultramarine, lead white	Lithopone and/or barium white and zinc white, ultramarine, chalk, oil
		Green	3	**C, O**, Na, Al, S, Cl, Ti, Zn, Ba, (Fe, Ca, Si, Sr, Pb, Cu)	Ultramarine, lithopone and/or barium white and zinc white, titanium white, yellow iron-rich earth pigment, chalk, lead white, phthalocyanine green, lead white	
		Green	7	**C, O, Zn**, Ca, Na, S, Ti, Fe, Al, Si, (Ba, Mg, Cl, K)	Lithopone and/or barium white and zinc white, chalk, ultramarine, titanium white, yellow iron-rich earth pigment	
		White	6	**C, O, Ti**, Zn, Ca, Al, Mg, Na, (S, Si, Fe, Cl)	Titanium white, zinc white, chalk, ultramarine, yellow iron-rich earth pigment	
*Mountains*, 2003-03306	1991	Brown	6	**C, O, Zn**, Ti, Fe, Ca, Na, Ba, Al, Mg, (Si, S, Pb, P)	Lithopone and/or barium white and zinc white, titanium white, red and yellow iron-rich earth pigment, lead white, bone black	
		Blue	1	**C, O, Zn**, Ca, Na, Ti, (Mg, Al, Ba, Si, Pb, S)	Lithopone and/or barium white and zinc white, chalk, titanium white, ultramarine, lead white	
		Violet	5	**C, O, Zn**, Ti, Ba, Al, Ca, Na, Si, S, (Mg, Fe, Pb)	Lithopone and/or barium white and zinc white, titanium white, chalk, ultramarine, yellow iron-rich earth pigment, lead white	
		Green	8	**C, O, Ca, Zn**, S, Na, (Si, Cl, Mg, Al, Fe, Ti)	Chalk, zinc white, ultramarine, yellow iron-rich earth pigment, titanium white	Yellow iron-rich earth pigment, Hansa yellow G, chalk, zinc soap, oil
		Green	9	**C, O, Ca, Zn**, S, Na, (Cl, Si, Mg, Fe, Al, Ba)	Chalk, lithopone and or barium white and zinc white, ultramarine, yellow iron-rich earth pigment	Chalk, yellow iron-rich earth pigment, Hansa yellow G, zinc soap, oil
		Green	10	**C, O**, Ca, Zn, Fe, S, Ba, Si, Cl, Al, (Ti, Na, Mg, Cu, Pb)	Chalk, lithopone and/or barium white and zinc white, yellow iron-rich earth pigment, ultramarine, titanium white, phthalocyanine green, lead white	Chalk, lithopone and/or barium white and zinc white, yellow iron-rich earth pigment, phthalocyanine green, Hansa yellow G, zinc soap, oil
		Grey	4	**C, O, Ti**, Zn, Ca, Ba, Al, Mg, (Na, S, Si, Fe)	Titanium white, lithopone and/or barium white and zinc white, chalk, ultramarine, yellow iron-rich earth pigment	
*Mount Huangshan*, 2003-03376	1993	Brown	6	**C, O, Zn**, Ti, Fe, Na, Ca, Al, Mg, Si, (S, P)	Zinc white, titanium white, yellow and red iron-rich earth pigment, bone black	
		Brown	7	**O, C, Fe**, Zn, Ca, Si, Al, Na, P, Mg, Ti, (Mn, Pb, Sr, S, Cl, K)	Yellow and red iron-rich earth pigment, umber, bone black, titanium white, lead white	
		Yellow	4	**C, O**, Ca, Ba, Cd, S, Zn, (Fe, Si, Al, Na, Mg, Cl, Ti, Pb)	Lithopone and/or barium white and zinc white, chalk, cadmium yellow or its variant, yellow iron-rich earth pigment, titanium white, lead white	
		Blue	2	**C, O, Zn**, Na, Ti, Ca, Al, Co, (Mg, Si, Fe, S)	Zinc white, ultramarine, titanium white, chalk, cobalt blue, yellow iron-rich earth pigment	Ultramarine, cobalt blue, chalk, zinc soap, oil
		Blue	8	**C, O**, Ti, Ca, Zn, Cr, Al, Pb, Mg, (Si, Na, Co, S)	Titanium white, chalk, zinc white, viridian, lead white, ultramarine, cobalt blue	
		Green	5	**C, O**, Cr, Zn, Ca, Ba, Ti, Fe, Si, Al, S, (Mg, Cd, P, Cl)	Viridian, lithopone and/or barium white and zinc white, titanium white, yellow iron-rich earth pigment, cadmium yellow or its variant, bone black	
		Grey	3	**C, O, Ti**, Zn, Ca, Al, Mg, (Na, S, Pb, Si)	Titanium white, zinc white, chalk, ultramarine, lead white	
*Mount Huangshan*, 2003-03307	1994	Brown	4	**C, O**, Ti, Fe, Zn, Ca, Mg, Al, (Na, Si, S, Pb, P)	Titanium white, yellow and red iron-rich earth pigment, zinc white, bone black, ultramarine, lead white	Iron-rich earth pigment, chalk, zinc soap, oil
		Brown	6	**C, O**, Fe, Ca, Si, Ti, Ba, S, Al, (Zn, Mg, P, Sr, Cl, Pb)	Red iron-rich earth pigment, bone black, lithopone and/or barium white and zinc white, lead white	
		Yellow (yellow cluster)	7	**C, O, Fe, Ca**, Zn, (Na, Mg, Si, S, Al)	Yellow iron-rich earth pigment, chalk, zinc white	
		Yellow (green cluster)		**C, O, Fe**, Ca, Zn, Al, (Si, Ti, Mg, S, Co, Ba, Cl)	Yellow iron-rich earth pigment, lithopone and/or barium white and zinc white, chalk, ultramarine, Co-containing pigment	
		Blue	2	**C, O**, Ti, Zn, Ca, Al, Ba, Mg, Co, (Si, Na, S)	Titanium white, lithopone and/or barium white and zinc white, chalk, ultramarine, cobalt blue	Lithopone and/or barium white and zinc white, chalk, ultramarine, cobalt blue, zinc soap, oil
		Green	3	**C, O, Ca**, Al, Ti, Cr, Zn, Si, (Fe, Mg, Co, Cl, Na, Sr, S, Pb, P)	Ultramarine, titanium white, viridian, zinc white, cobalt blue, yellow iron-rich earth pigment, lead white, bone black	Chalk, ultramarine, viridian, cobalt blue, chalk, zinc soap, oil
		Green	5	**C, O, Zn**, Fe, Na, (Cl, Ca, Al, Si, Ti, S)	Zinc white, yellow iron-rich earth pigment, ultramarine, chalk, titanium white	
		Grey	1	**C, O, Zn**, Ti, Na, Ca, Mg, Al, (S, Si, Co, Fe)	Zinc white, titanium white, chalk, ultramarine, cobalt blue contamination, yellow iron-rich earth pigment	
*Mount Huangshan*, 2003-03378	1995	Brown	8	**C, O, Zn**, Ca, Na, Fe, Si, (P, Al, Mg, S, Ti, Cr, Mn, Ba, K, Cl)	Lithopone and/or barium white and zinc white, bone black, umber, titanium white, Cr-containing yellow pigment, titanium white	
		Blue	1	**C, Zn, O**, Al, Ca, Co, Na, (Ti, Mg, Si, S, Ba)	Lithopone and/or barium white and zinc white, chalk, ultramarine, cobalt blue, titanium white	Chalk, ultramarine, cobalt blue, oil
		Blue	2	**C, O, Zn**, Ti, Ba, Ca, Al, Na, Mg, (Cr, Si, S, Fe)	Lithopone and/or barium white and zinc white, titanium white, chalk, ultramarine, viridian	
		Violet	5	**C, O**, Zn, Ti, Ca, Fe, Ba, Al, Si, Mg, (Na, P, S, K)	Lithopone and/or barium white and zinc white, titanium white, red iron-rich earth pigment, ultramarine, bone black	
		Green	3	**C, O, Zn**, Fe, Na, Ca, (Ti, Al, Ba, Cl, Si, Mg, S)	Lithopone and/or barium white and zinc white, chalk, titanium white, ultramarine, yellow iron-rich earth pigment, chalk	Hansa yellow G, yellow iron-rich earth, chalk, oil
		Green	4	**C, O, Cr**, Ca, Ba, Zn, Si, (Al, S, Mg, Na, Cl, Ti, Fe, K)	Viridian, chalk, lithopone and/or barium white and zinc white, titanium white, yellow iron oxide	
		Grey	7	**C, O**, Ti, Zn, Ba, Ca, Al, Mg, (Na, Fe, S, Pb, Si, Cr)	Titanium white, lithopone and/or barium white and zinc white, chalk, ultramarine, yellow iron-rich earth pigment, viridian, lead white	
*Mountain*, 2003-03293	1995	Brown	5	**C, O**, Ti, Fe, Ca, Zn, Al, (Mg, Na, Si, S, P)	Titanium white, yellow iron-rich earth pigment, bone black, zinc white	Chalk, yellow iron-rich earth pigment, bone black, zinc soap, oil
		Brown	8	**O, C, Fe**, Ca, Si, P, Al, Mg, Mn, Zn, (Na, S, K, Ti, Sr, Pb, Cl)	Red iron-rich earth pigment, umber, bone black, zinc white, titanium white, lead white	
		Blue	3	**C, O**, Zn, Ti, Ca, Na, Mg, (Al, Cr, S, Si, Cl, Pb)	Zinc white, titanium white, chalk, ultramarine, viridian, lead white	
		Green	6	**C, O**, Zn, Ti, Ca, Cr, Na, Fe, Al, (Mg, Si, S, Cl, Sr)	Zinc white, titanium white, chalk, viridian, yellow iron-rich earth pigment	
		Green	7	**C, O, Cr**, Ca, Ba, Si, Al, Zn, Ti, (S, Na, Mg, Cl)	Viridian, chalk, lithopone and/or barium white and zinc white, titanium white	Viridian, chalk, lithopone and/or barium white and zinc white, Hansa yellow G, oil
		Grey	4	**C, O, Ti**, Zn, Ca, Al, Mg, (Na, Co, S, Cr, Si, Fe)	Titanium white, zinc white, chalk, ultramarine, viridian, Co-containing pigment, yellow iron-rich earth pigment	
*Mount Huangshan*, 2003-03257	1996	Brown	8	**C, O, Zn**, Ba, Fe, Pb, Ca, S, Na, Al, (Si, Cr, Cl, P, Sr, Mg)	Lithopone and/or barium white and zinc white, Prussian blue, Cr-containing yellow(s), organic red on Al-containing substrate, ultramarine, bone black	Lithopone and/or barium white and zinc white, Prussian blue, chrome yellow, yellow iron-rich earth pigment, organic red, bone black, zinc soap, oil
		Yellow	7	**C, O, Fe**, Ca, Ba, Cd, S, Zn, (Cl, Sn, Mg, Na, Al, Si)	Yellow iron-rich earth pigment, chalk, lithopone and/or barium white and zinc white, cadmium yellow or its variant, organic red on Sn-containing substrate	Yellow iron-rich earth pigment, lithopone and/or barium white and zinc white, cadmium yellow or its variant, naphthol red AS-D, oil
		Blue	2	**C, O, Ti, Zn**, Ca, Na, (Fe, Si, Mg, Pb, Al, S, P, Cl)	Titanium white, zinc white, chalk, yellow iron-rich earth pigment, lead white, ultramarine, bone black	
		Blue	3	**C, O**, Ti, Zn, Ca, Ba, Fe, Al, Mg, (S, Si, Cl, Cr, Na, Pb)	Titanium white, lithopone and/or barium white and zinc white, chalk, Prussian blue, ultramarine, viridian, lead white	
		Green	5	**C, O, Zn, Ca**, S, Na, Ba, (Mg, Cl, Al, Si, Sr, P, Ti)	Lithopone and/or barium white and zinc white, ultramarine, bone black, titanium white	Lithopone and/or barium white and zinc white, chalk, Hansa yellow G, ultramarine, yellow iron-rich earth pigment, zinc soap, oil
		Green	6	**C, O, Zn, Ba**, Pb, S, Fe, Cr, Na, Ca, (Al, Si, P, Mg, Cl, K)	Lithopone and/or barium white and zinc white, chrome yellow, Prussian blue, bone black	Lithopone and/or barium white and zinc white, chrome yellow, Prussian blue, chalk, zinc soap, oil
		Grey	4	**O, C, Ti**, Zn, Ca, Ba, Na, (Mg, Si, P, Al, Pb, S)	Titanium white, lithopone and/or barium white and zinc white, ultramarine, bone black, lead white	

* Major elements are provided in bold type, minor elements in plain type and trace elements in brackets.
